# Data-Limited Population-Status Evaluation of Two Coastal Fishes in Southern Angola Using Recreational Catch Length-Frequency Data

**DOI:** 10.1371/journal.pone.0147834

**Published:** 2016-02-01

**Authors:** Jennifer Beckensteiner, David M. Kaplan, Warren M. Potts, Carmen V. Santos, Michael R. O’Farrell

**Affiliations:** 1 UMR 212 EME, Centre de Recherche Halieutique Méditerranéenne et Tropicale, Institut de Recherche pour le Développement (IRD), Sète, France; 2 Department of fisheries, Virginia Institute of Marine Science (VIMS), College of William & Mary, Gloucester Point, Virginia, United States of America; 3 Department of Ichthyology and Fisheries Science, Rhodes University, Grahamstown, South Africa; 4 Faculdade Ciêncas da Universidade Agostinho Neto (FCUAN), Luanda, Angola; 5 Department of Wildlife, Fish and Conservation Biology, University of California, Davis, Davis, CA, United States of America; Virginia Commonwealth University, UNITED STATES

## Abstract

Excessive truncation of a population’s size structure is often identified as an important deleterious effect of exploitation, yet the effect on population persistence of size-structure truncation caused by exploitation is often not quantified due to data limitations. In this study, we estimate changes in eggs per recruit (EPR) using annual length-frequency samples over a 9 year period to assess persistence of the two most important recreational fishes in southern Angola: west coast dusky kob (*Argyrosomus coronus*) and leerfish (*Lichia amia*). Using a length- and age-structured model, we improve on an existing method to fit this type of model to length-frequency data and estimate EPR. The objectives of the methodological changes are to add flexibility and robustness to the approach for assessing population status in data-limited situations. Results indicate that dusky kob presents very low levels of EPR (5%-10% of the per recruit reproductive capacity in the absence of fishing) in 2013, whereas large inter-annual variability in leerfish estimates suggest caution must be applied when drawing conclusions about its exploitation status. Using simulated length frequency data with known parameter values, we demonstrate that recruitment decline due to overexploitation leads to overestimation of EPR values. Considering the low levels of EPR estimated for the study species, recruitment limitation is not impossible and true EPR values may be even lower than our estimates. It is, therefore, likely that management action, such as the creation of Marine Protected Areas, is needed to reconstitute the west coast dusky kob population.

## Introduction

Fishing alters the age and size structure of fish populations, as well as affects population persistence and robustness to environmental fluctuations [1–5]. One of the immediate effects of fishing is a reduction in the mean size of fish in the population [6], resulting in demographic changes in individual and population-wide reproductive outputs [7]. Severe overfishing can eventually lead to population decline and collapse due to recruitment limitation [8,9]. In order to identify population overexploitation and quantify benefits from different management options, there is a need to estimate indicators of the impact of exploitation on fish stocks [10]. Here, we present estimates of temporal changes in population persistence based on per-recruit measures of the reproductive capacity of two fishes derived from catch length-frequency distributions in a recreational fishery in southern Angola. The methodology used is a general adaptation of statistical catch-at-length and/or catch-at-age models [11–13] and has wide applicability to fisheries assessment and management in data limited situations. To facilitate its use, we provide generic Matlab® code implementing the method.

Eggs-per-recruit (EPR = LEP, Lifetime egg production, often taken to be proportional to SSBR, Spawning stock biomass per recruit) is the number of eggs that an average recruit produces over its lifetime calculated as the sum over all ages of reproductive output at a given age multiplied by the probability of survival from recruitment to that age [13–15]. EPR quantifies how much each individual contributes to replacing itself in the next generation via reproduction; hence it can be used to quantify the tendency of a population to persist. When it is combined with estimates of recruitment as a function of egg production (e.g., a stock-recruitment relationship) or, at a minimum, limit reference points for egg production needed to avoid population collapse, it can be used to infer population persistence status [16,17]. Specifically, a population with age-structure and intra-cohort density-dependent recruitment will collapse if the lifetime replacement rate is less than 1, where lifetime replacement rate is the product of EPR and the slope of the egg-recruit relationship at the origin [8,18]. As such, accurately estimating EPR is extremely valuable for assessment of exploited fish populations.

One method of estimating EPR in data limited situations is to fit estimated catch length-frequency from a length- and age-structured population model to observed catch length-frequency data [13,19,20]. Optimization is used to find the fishing mortality rate that maximizes the correspondence between these two, and this fishing mortality rate is in turn used to estimate EPR for the population [13]. EPR is then expressed as a fraction of the natural, unfished EPR, i.e. FNEPR (= FLEP, Fractional lifetime egg production = SPR, Spawning potential ratio), so that values can be compared with those from more data rich species, for which minimal values of FNEPR necessary to avoid population collapse are better known. This methodology provides a relatively straightforward approach to stock assessment in small-scale fisheries because it requires less data than traditional age-structured assessment models and is under certain conditions less sensitive to sources of uncertainty, such as the value of the natural mortality rate [13]. Given data limitations in the recreational fisheries of southern Angola that are the focus of this study, a full stock assessment is unrealistic and FNEPR calculation is an appropriate approach.

A number of specific methodologies for estimating fishing mortality rates and EPR from catch length-frequency data following the general procedure described above have been developed [11,21,22]. Hordyk *et al*. [20] developed a length-based Spawning-Per-Recruit (LB-SPR) model assuming equilibrium state and constant recruitment, and used simulations and a case study to test its ability to estimate fishing mortality relative to natural mortality and SPR (aka FNEPR). Klaer *et al*. [19] used an assessment method based on the average length of catch to evaluate the efficacy of target- and limit-based harvest control rules in terms of achieving specific long-term management objectives for several Australian demersal temperate trawl species. Finally, O’Farrell & Botsford (2006) [23] fit an age- and size-structured model to catch length-frequency data from the California rockfish fishery and then compared FNEPR estimates early on in the development of the fishery with more recent estimates to assess persistence status in five rockfish species.

**Fig 1:**
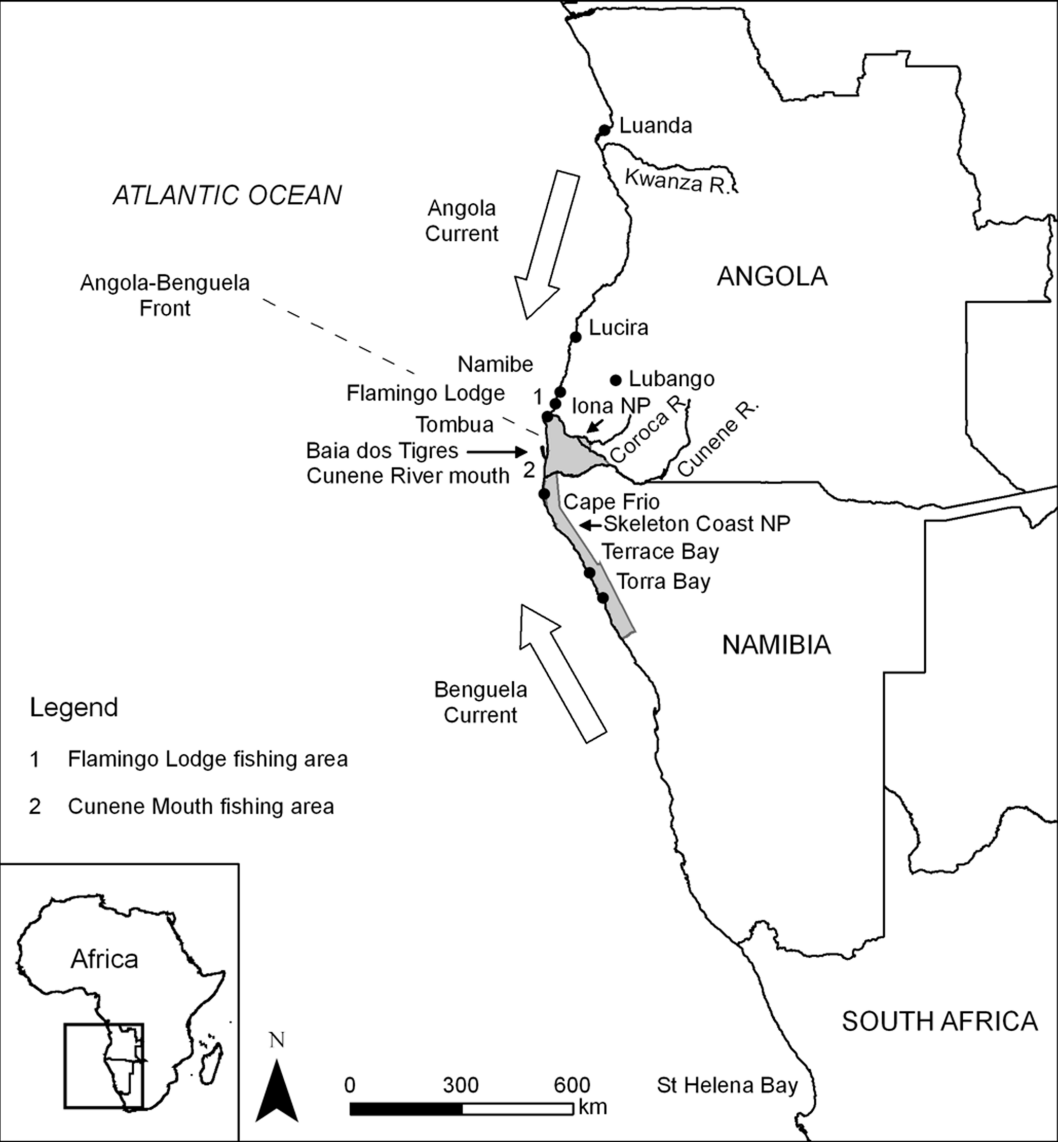
Map of the study area. Map of the south-western coast of Africa showing the study area, Flamingo Lodge (1) and sites mentioned in the text. Namibian land adjacent to the coast just south of the Cunene River mouth (2) are protected as part of the Skeleton Coast National Park, whereas lands north of the Cunene River form the Angolan Iona National Park (grey areas). Adapted from Potts *et al*. 2010.

Angola is a large country with a coastline of 1,650km, comprising extremely rich marine resources [24,25]. The marine environment in this region is influenced by two major ocean currents, the northward flowing cold Benguela current and the southward flowing warm Angola current [26], and benefits from high productivity due to strong upwelling at the frontal zone between these two currents [27–29]. Following 27 years of civil war that ended in 2002, the inland population has recently undergone large-scale shifts to the coastal towns [25]. Coastal fisheries in Angola comprise a significant, growing artisanal fishery, a large subsistence fishery and a growing local and foreign recreational fishery. Recreational fisheries in particular have the potential to substantially contribute to the local economy of the southern Angolan region [30]. If left unregulated, these fisheries may experience significant declines, such as have been observed for similar fisheries in Namibia and South Africa [30–32] As such, it is important to evaluate fishing impacts now before declines occur. At present, the majority of fishing activity in southern Angola is centered between the towns of Namibe and Tombua (Fig 1), with the surrounding areas largely exempt from fishing pressure with the exception of the Cunene River mouth. The relatively recent expansion of fishing activity and the existence of untouched marine areas provide an opportunity to collect pre-impact state indicators and to implement conventional and spatial management strategies before heavy exploitation occurs.

Here, we develop a length- and age-structured population model for the two most important recreational species in southern Angola, and apply it to the assessment of fishing pressure and FNEPR of these stocks. Our methodology is based on that of O’Farrell & Botsford [13], but we have included a number of methodological improvements designed to increase the robustness of estimates. In addition, we examine a number of different sources of uncertainty and bias in model estimates, such as recruitment limitation and variability [33]. Finally, we calculate temporal trends in fishing mortality and per capita egg production for the two species, and use these to assess their conservation status and necessity of management action.

## Materials and Methods

### Ethics Statement

Permission to collect samples was obtained from the Namibe director of the Angolan Ministry of Fisheries (Jorge Martins), through the Department of Biology at the University of Agostinho Neto, which complied with relevant regulations. As all samples were taken from already dead specimens either captured as part of the recreational fishing activities at Flamingo Lodge (15°34’13.02’S, 12°01’07.53”) or purchased in the local markets, including the Namibe Harbor market (15°11’50.62’S, 12°08’06.76”), Praia Pinda beach market (15°44’06.59’S, 11°54’38.07”) and Tombua beach market (15°47’57.49’S, 11°51’17.70”), no ethics approval was necessary. The purchased fish were all captured in the local artisanal fishery and therefore did not have a lot number.

### Data

We examined the temporal evolution of catch length-frequency from the onset of extensive exploitation until the present for a Sciaenid, the west coast dusky kob (*Argyrosomus coronus*, Griffiths & Heemstra 1995 [34]) and a Carangid, the leerfish (*Lichia amia*, Linnaeus 1758). Relatively little biological research has been carried out on these teleost species, though basic biological information (growth, reproduction and mortality parameters) are available for the stocks of southern Angolan [35,36]. Both species are exploited by recreational shore-based anglers, with each species individually representing roughly a quarter of the overall catch in terms of biomass [37], as well as by subsistence and artisanal fisheries, including artisanal line, illegal gillnet and beach-seine fisheries.

Data for this study were gathered as part of a tourist-based recreational fishery monitoring program from 2005 to 2011 (May—October fishing season) at Flamingo lodge in Southern Angola (Fig 1; Table A in S3 File). This fishing area comprises a 28.2 km stretch of uninhabited coastline. Monitoring took place whenever fishing took place at the lodge. Hook sizes ranged from 1/0 to 8/0 for dusky kob, and 5/0 to 8/0 for leerfish. Once captured, fishes were measured (fork and total lengths) and weighed. Dusky kob were separated into 50 mm catch size bins, each representing approximately one year of growth (based on the estimated von Bertalanffy growth curve). This produced 32 catch size classes from 0 to 1500 mm Total Length (TL). Leerfish were separated into 100 mm catch size bins, again representing approximately one year of growth, yielding at total of 11 catch size classes from 200 to 1200 mm Fork Length (FL).

Growth parameters, natural mortality rates and length-at-50% sexual maturity were estimated following the methodology of Potts *et al*. (2008, 2010) [35,36] (see Table 1 for parameter values).

**Table 1:** Parameter set used to generate length frequency data. Fish lengths are expressed in mm fork length for the leerfish (*A*.*Limia*) and mm total length for the dusky kob (*A*.*Coronus*).

Parameter definition (unity)	Parameter	Leerfish	Dusky kob
***Biological parameters***			
Asymptotic von Bertalanffy length (mm) ^a^	L_∞_	1065.37	1856.1
Coefficient of variation of asymptotic length	CV L_∞_	0.10	0.10
Age at which individual would have been length = 0 (y) ^a^	t_0_	-1.16	- 1.75
Von Bertalanffy growth parameter (y^-1^) ^a^	k	0.31	0.11
Natural mortality rate (y^-1^) ^a^	M	0.28	0.12
Length—egg relationship ^a^	a	1.00e-09	4.00e-09
Length—egg relationship ^a^	b	3.36	3.14
Length at 50% maturity/ maturity inflection point (mm)	μ_mat_	626.13	928.86
Maturity ogive width (mm)	σ_mat_	102.13	121.81
Length at the start of the selection (mm)	L_S_	200^b^	250^b^
Length at the end of the selection (mm)	L_E_	900^b^	1300^b^
***Structural parameters for the model***			
Number of timesteps per year	num_ts	2	2
Maximum age (year)	max_age	11	11
Number of size classes	NS	61	61

^a^ Adapted from Potts *et al*. 2008, 2010.

^b^ Values indicated are for the “all years” hypothesis. For “year-by-year” calculations, values varied between years, with L_S_ ranging from 200 to 600 mm FL for leerfish and 250 to 550 mm TL for dusky kob, and L_E_ ranging from 800 to 900 mm FL for leerfish and 800 to 1300 mm TL for dusky kob.

### Age- and length-structured model

The length- and age-structured population model we develop here is based on that of O’Farrell & Botsford (2005) [13], the principal equations of which are included here. A von Bertalanffy growth curve was used to describe the growth of individual fish. The impact of individual-level variation in growth on model estimates of the length-frequency distribution was assessed by assuming that individual maximum asymptotic lengths, L_∞_, are distributed according to a gamma distribution whose mean is the observed most probable value for L_∞_ [38]. As empirical estimates of the coefficient of variation of the asymptotic length parameter were subject to large uncertainties, we decided to assume it was 0.1 for both species and then later assess sensitivity of population status estimates to the precise value of this parameter. Individuals were divided into length classes, length-at-age for each of which was represented by:

$${\text{L}}_{\text{a},\text{i}}={\text{L}}_{\infty ,\text{i}}\;\left(1-{\text{e}}^{\left(-\text{k}\left(\text{a}-{\text{a}}_{0}\right)\right)}\right)$$ (1)

where L_a,i_ is fish length at age a for individuals in the i^th^ length class, L_∞,i_ is asymptotic length for individuals in the i^th^ length class, k is growth rate, and a_0_ is the theoretical age at which the length of a fish equals zero. A total of 61 length classes (not to be confused with the catch size classes above; these length classes are for asymptotic maximum size) were used for each species. Width of length classes varied in relation to the gamma distribution, being smaller close to the mean L_∞_ and larger away from it, so that each length class represented approximately 1/61^th^ of the total population at recruitment. Survival of individuals from one age class to the next was estimated by:

$${\text{N}}_{\text{a}+1,\text{i}}={\text{N}}_{\text{a},\text{i}}.\text{e}\;{(}^{-\left(\text{M}+{\text{S}}_{\left({\text{L}}_{\text{a},\text{i}}\right)}.\text{F}\right)})$$ (2)

where N_a,i_ is the number of individuals at age a in length class i, M is the natural mortality, F is the fishing mortality, and S_L_ is the selectivity at length L.

Fishing selectivity as a function of length was given by a logistic curve:

$${\text{S}}_{\text{L}}=\frac{1}{1+{\text{e}}^{-\frac{\left(\text{L}-\mu_{\text{S}}\right)}{\sigma_{\text{S}}}}}$$ (3)

where μ_S_ is the inflection point of the selectivity logistic curve and σ_S_ is the width of the transition from 0 to full selectivity. The expected number of individuals caught in each age-size class was estimated using:

$${\text{C}}_{{\text{L}}_{\text{a},\text{i}}}={\text{N}}_{{\text{L}}_{\text{a},\text{i}}}.\left[1-\;{\text{e}}^{-\;\left(\text{M}+{\text{S}}_{{\text{L}}_{\text{a},\text{i}}}.\text{F}\right)}\right].\frac{{\text{S}}_{{\text{L}}_{\text{a},\text{i}}}.\text{F}}{\text{M}+{\text{S}}_{{\text{L}}_{\text{a},\text{i}}}.\text{F}}$$ (4)

Catch in age-size classes having similar sizes were binned together according to the previously established catch size-classes to obtain the final model prediction of catch length-frequency as a function of fishing mortality parameters.

#### Calibration of fishing mortality parameters

Model predictions of the catch length-frequency distribution were normalized in order to represent the same total number of individuals as in the observed catch length-frequency distribution for a given year, and maximum likelihood estimates (MLE) of F, μ_S_, and σ_S_ (Table 1) were obtained via an iterative process by minimizing the Kullback-Leibler (KL) divergence (also referred to as the separation statistic) [39]:

$$\theta \;\left(\text{F},\mu_{\text{S}},\sigma_{\text{S}}\right)=\sum_{{\text{L}}_{\text{S}}}^{{\text{L}}_{\text{max}}}{\text{O}}_{\text{L}}\;.\text{ln}(\frac{{\text{O}}_{\text{L}}}{{\text{P}}_{\text{L}}})$$ (5)

where O_L_ and P_L_ are the observed and predicted number of individuals caught at length L, respectively. The KL divergence is equivalent to the multinomial negative log-likelihood for the observed number of individuals in each size class given the population model (up to a constant that is independent of model parameters [39]). It is an appropriate measure for quantifying the difference between frequency distributions [39,40]. Eq 5 was calculated by summing over length classes with a non-zero number of observed individuals [39], and minimized using the Nelder–Mead simplex algorithm, implemented with the MATLAB (Math Works 2002) library function “*fminsearch*”.

Though this basic approach was used on each year’s data to estimate fishing mortality parameters, the global minimization process (i.e., taking into account all years for which data was available) was in some cases modified to link parameter estimations from different years. Four different global minimization approaches were used. In the first, no such linkage between years was used and independent values of fishing pressure and selectivity parameters were estimated for each year separately, treating each annual length-frequency distribution as if it represents the long-term equilibrium distribution for a constant selectivity and fishing pressure [13]. This approach, referred to here as the “year-by-year” hypothesis with equilibrium conditions, is likely to have the best fit to annual length-frequency distributions, but the model has 3 free parameters per year, and, therefore, may be underdetermined.

The second approach was a modification of the first to assume that selectivity parameters (essentially a function of the gear used and the fishing methodology) remain constant over time. This approach has the advantage of reducing the number of free parameters in the model, and may be justified if fishing methodology does not change significantly over time. For this approach, referred to as the “all years” hypothesis, parameter estimation is carried out simultaneously for all years with a single KL divergence calculated as the sum of KL divergences for each individual year given a (universal) pair of selectivity parameters and individual fishing rates for each year.

An alternative approach that integrates some aspects of the temporal evolution of fishing pressure was also considered. In this approach, we estimated parameters assuming that fishing began in 2005, referred to here as the “n-fishing years” hypothesis. This is roughly correct for the southern Angolan stocks that were lightly exploited before this time. For this model, estimated values of fishing mortality F represent the average fishing pressure from the beginning of the fishery to the year being examined.

Collectively, these different hypotheses lead to 4 different approaches for estimating fishing pressure (Table 2):

- using the “year-by-year” hypothesis and assuming equilibrium conditions, as in the original model in O’Farrell & Botsford (2005) [13];
- treating selectivity as constant, i.e., “all years”, and assuming equilibrium conditions;
- treating selectivity as “year-by-year”, but using “n-fishing years”;
- both “all years” and “n-fishing years” assumptions.

**Table 2:** Description of the four approaches used to estimate exploitation parameters according to different hypotheses.

		SELECTIVITY	
		Independent value each year	Constant over time
**EVOLUTION**	Equilibrium	Approach i ^a^	Approach ii
**OF FISHING**		Year-by-year / equilibrium	All years / equilibrium
**PRESSURE**	Fishing began in 2005	Approach iii	Approach iv ^b^
** **		Year-by-year / n-fishing years	All years / n-fishing years

^a^ the original approach as O’Farrell and Botsford (2005).

^b^ the new approach.

The four approaches are necessary and complementary to each other because they address the long-term change in population persistence (estimation of which is the principal goal of this manuscript) in different ways. Approaches iii and iv respond immediately to changes in population size structure due to the onset of fishing pressure, and, therefore, provide an immediate diagnostic of the average fishing rate since the beginning of the fishery. Due to the assumption of quasi-equilibrium conditions, approaches i and ii only identify population changes after multiple years of fishing, but have the advantage of showing a decline in population status over time. The quasi-equilibrium assumption is unrealistic, but observing this decline in status is useful for identifying population trends, particularly given model uncertainties that may lead to poor estimation of the absolute value of the fishing rate. This approach of using multiple different model structures to assess population status is consistent with the ensemble approach to stock assessment [41].

An additional approach whereby a time series of instantaneous fishing rates (as opposed to the average fishing rate since the onset of fishing) was also considered, but is not presented here because the flexibility of this approach led to strong inter-annual variability in instantaneous fishing mortality rates that was not suitable for estimation of long-term FNEPR values.

#### Eggs-per-recruit estimation

Egg production as a function of fish age or size must be known to calculate EPR. As this was not known for our study species, fish weight was used here as a proxy for individual egg production. EPR (here assumed proportional to spawning stock biomass per recruit, SSBR) was calculated by multiplying fish weight by the fraction of mature individuals in a given age-size class and survival to that age-size class, and then summing over all age-size classes:

$$\text{EPR}=\frac{\sum_{\text{i}}\sum_{\text{at}}\;{\text{N}}_{\text{a},\text{i}}\;.{\text{m}}_{{\text{L}}_{\text{a},\text{i}}}\rho_{{\text{L}}_{\text{a},\text{i}}}}{\sum_{\text{i}}\;{\text{N}}_{0,\text{i}}}$$ (6)

where N_a,i_ is the number of individuals surviving to age a in length class i (N_0,i_ being the number of age-0 recruits to the population in that length class), $m_{L_{a,i}}$ is the fraction of mature individuals and $\rho_{L_{a,i}}$ is fish weight. The fraction of mature individuals in an age-size class was modeled as a logistic function of length analogous to Eq 3. Fish weight was assumed to be an allometric function of fish length: ρ_L_ = aL^b^, where ρ_L_ is weight at length L, and a and b are the weight-length relationship coefficient and exponent, respectively.

Though our methodology calculates a different fishing mortality rate for each year of data, we treated the estimated fishing mortality as if it was constant in time when calculating EPR. Therefore, EPR estimates represent the long-term equilibrium value that will occur *if* fishing mortality is indefinitely held constant at the value estimated for a specific year, consistent with our objective of evaluating long-term population persistence.

Finally, the fraction of natural EPR (FNEPR) was estimated by dividing the calibrated EPR value by the value of EPR when F = 0. Where appropriate, FNEPR values in a given year are compared with those for the initial year of data to evaluate declines in population reproductive capacity.

Matlab code for parameter estimation and FNEPR calculations can be found in S1 File.

### Evaluation and performance of the model

It has been previously observed that a good fit to the data by the model described above can be obtained at times by several different sets of parameter values with approximately the same likelihood [13]. When maximum likelihood estimates are not unique, the set of parameters is said to be not identifiable [42]. Lack of identifiability produces large parameter value uncertainties and potentially implausible parameter estimates. To assess problems of identifiability in our model, as well as to determine if using the “all years” hypothesis, which has a smaller number of free parameters, reduces these problems, we plotted KL divergence surfaces as a function of pairs of parameters (e.g., F and μ_S_), and looked for highly-elongated, diagonal ellipsoidal contours around the minimum indicating a problem of identifiability between the two parameters in question.

Uncertainty in parameter estimates was assessed using a bootstrap approach, resampling with replacement of the lengths of the individual fish captured, maintaining constant the total number of fish (varying between years: 15–180 with median 100 fish for dusky kob; 51–452 with median 106 fish for leerfish). This was done to generate alternative, statistically equivalent, catch length-frequency distributions. As this approach does not account for uncertainty in the non-estimated biological parameters, a sensitivity analysis was also performed on eight input parameters (growth, natural mortality, and maturity parameters). Sensitivity was calculated using the formula:

$${\text{S}}_{\text{p}}=\text{p}.\frac{\text{d}\left(\text{FNEPR}\right)}{\text{dp}}$$ (7)

where S is the sensitivity of FNEPR for a given year with respect to parameter p, p being one of the eight non-estimated input parameters. d(FNEPR) was calculated as the difference between FNEPR based on the best fit parameter values and FNEPR calculated with parameter p increased or decreased by 10% (i.e., dp/p = ±0.1). This procedure leads to two estimates of sensitivity, one for increasing p and another for decreasing p. Only the sensitivity estimate with the largest magnitude is reported here.

The sensitivities of fishing mortality with respect to the eight parameters were also calculated, results for which are presented in Fig F in S2 File.

#### Simulations to test robustness of approach

The estimation approach described above assumes that recruitment is approximately constant and unaffected by fishing, but this may not be the case for variable environments and heavily exploited populations. We, therefore, carried out four sets of simulations to test the impact of stochastic and deterministic variability in recruitment and small sample size on fishing mortality and FNEPR estimates for dusky kob (leerfish produced similar simulation results). In all cases, the simulated fish population was first initiated by simulating 50 years in the absence of fishing. Then another 50 years was simulated at a constant, fixed fishing rate (chosen so as to be close to the value obtained from applying the assessment method to real data; F = 0.5, μ_S_ = 792 mm TL, σ_S_ = 111 mm TL). The simulated fish population dynamics was identical to the population dynamics assumed in the assessment method itself, except that recruitment rates could be controlled and modified.

The four sets of simulations explored were:

Simulation 1: “Transient phase”: We generated the transient size structure resulting from a change in fishing mortality F, analogous to the instantaneous development of a new fishery. Recruitment was held constant in these simulations.

Simulation 2: “Recruitment limitation”: Next, the impact of recruitment limitation during the transition from a virgin to a fished population state was examined. Recruitment was allowed to vary between timesteps and its value was governed by the Beverton-Holt stock-recruitment relationship [11]. The stock-recruitment relationship was parameterized so that the population collapses for any fishing rate that reduces FNEPR below 0.35 (corresponding to a steepness value of 0.42) [18]. As the simulated fishing rate produces a FNEPR of 0.041, the population collapsed, allowing us to examine the effect of recruitment limitation due to overfishing on parameter estimates during the initial years of the fishery.

Simulation 3: “Recruitment stochasticity”: The estimation method was tested on data generated with random recruitment variability. Recruitment variability was generated by randomly drawing recruitment values for each timestep from a gamma distribution with a fixed mean and coefficient of variation of 0.2. Recruitment values were not linked to the size of the adult population, so this simulation specifically assessed the impact of random recruitment variability, as opposed to the previous simulation which addressed deterministic changes in the level of recruitment.

Simulation 4: “Sample stochasticity”: Stochasticity in observed catch length-frequency distributions can also occur due to small sample size. To test the impact of small sample size on assessments, we randomly drew 100 fish from the theoretical predicted catch length-frequency distribution (which is equivalent to the length-frequency distribution for an infinite sample size). For these simulations, recruitment was held constant.

The last two simulations involving stochasticity used parameter estimation approach iv and were repeated 1000 times to assess variability in model results.

## Results

In the interest of brevity, not all results for all four parameter estimation approaches are presented below. Rather, we focus on those tendencies best illustrated by each of the approaches. When examining fishing pressure, we primarily use the “n-fishing years” approaches (approaches iii and iv) as F estimates using this method respond more quickly to the onset of fishing, whereas when examining FNEPR, we tend to use the equilibrium approaches (approaches i and ii) as this method more clearly reflects long term changes in population size structure. Similarly, the “all years” approach to estimating selectivity parameters (approaches ii and iv) is generally preferable when examining fishing rate time series as selectivity is held constant over time, whereas the “year-by-year” approach (approaches i and iii) is useful when assessing potential changes in fishing practice over time.

### Estimated mortality and FNEPR trends

**Fig 2:**
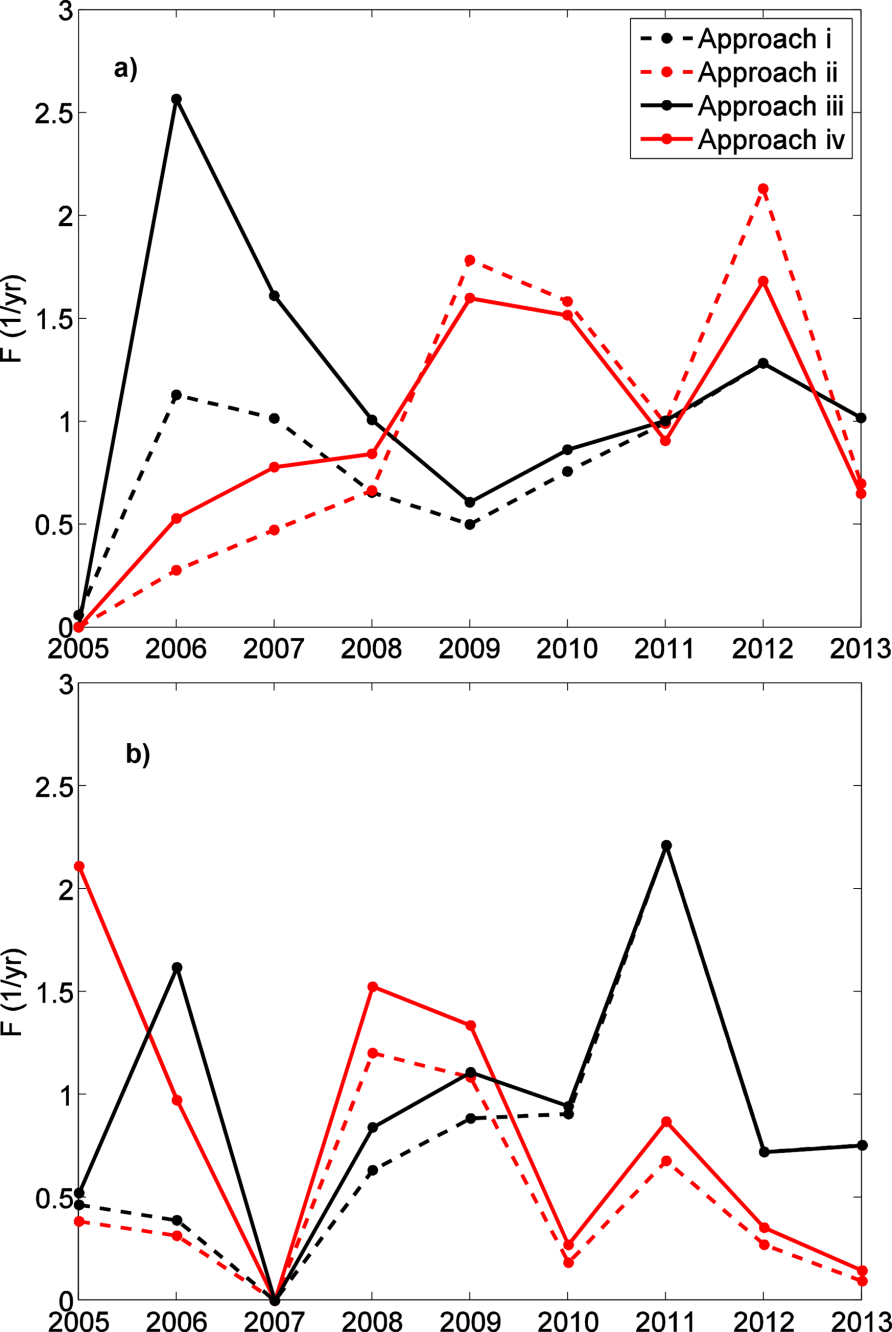
Estimation of fishing mortality rates based on the 4 parameter estimation approaches. Fishing mortality rates estimated for dusky kob (*A*. *coronus*) (a) and leerfish (*L*.*amia*) (b) according to different assumptions for global parameter minimization. Results assuming constant selectivity (μ_S_ and σ_S_ fixed over years) are presented in red (approaches ii and iv), whereas those assuming yearly selectivity parameters are in black (approaches i and iii). Dashed lines represent rates assuming the population is at equilibrium (approaches i and ii), whereas solid lines use the “n-fishing years” hypothesis (approaches iii and iv).

Assuming that fishing began in 2005 and that selectivity parameters remain constant for all years (parameter estimation approach iv), the two study species have rather different trends in exploitation and reproductive capacity over time. There was a general increase in the fishing mortality of the dusky kob since 2005 (with some variability), whereas the fishing mortality estimates for leerfish varied substantially, with a decreasing trend overall (Fig 2, solid red line). Fishing mortality for fully selected individuals was estimated to be 0.65 yr^-1^ for dusky kob, and 0.15 yr^-1^ for leerfish in 2013 (see Table 3 for estimates of selectivity parameters). Fishing mortality estimates for leerfish are consistently biased with respect to estimated uncertainty ranges for this parameter, with mortality estimates always being below the lower 25% quartile of bootstrap estimations (see Fig B in S2 File). Fishing mortality estimates are zero for 2005 for dusky kob and for 2007 for leerfish.

**Table 3:** Estimates of fishing mortality, selectivity parameters (inflection point and width of the selectivity curve µ_S_ and σ_S_ respectively) and KL divergences obtained for each assessment approach. AIC is the Akaike Information Criterion, calculated as AIC = 2 * KL divergence + 2 * number of parameters, where the KL divergence is calculated as the sum over all years of annual KL divergences. “Year-by-year” approach had 27 estimated parameters whereas “All years” had 11 free parameters. The units of selectivity parameters are mm total length for dusky kob (*A*.*Coronus*) and mm fork length for leerfish (*A*.*Limia*).

Species	Assumptions	Equilibrium		N-fishing year	
		year-by-year	all years	year-by-year	all years
		Approach i	Approach ii	Approach iii	Approach iv
Dusky kob	Selection μ_S_	809.6^a^	807.9	816.7^a^	791.9
	Selection σ_S_	121.7^a^	114.5	117.8^a^	110.7
	KL divergence	184.7	258.7	193.3	272.4
	AIC	411.4	535.4	428.6	562.8
	F 2013 (yr^-1^)	1.02	0.70	1.02	0.64
	FNEPR 2013	0.060	0.083	0.060	0.090
Leerfish	Selection μ_S_	698.9^a^	648.0	708.6^a^	684.3
	Selection σ_S_	72.5^a^	80.5	69.00^a^	85.9
	KL divergence	53.2	143.4	70.9	157.4
	AIC	148.4	304.8	183.7	332.8
	F 2013 (yr^-1^)	0.76	0.10	0.76	0.15
	FNEPR 2013	0.31	0.70	0.31	0.61

^a^ Mean of values’ series because selectivity parameters were estimated independently for each year.

Other parameter estimates are summarized in Table 3 for each of the different parameter estimation approaches used. Smaller values for the KL divergence (i.e., higher likelihoods) are obtained from the approach assuming changing selectivity over years (approaches i and iii); this is consistent with the larger number of free parameters to be estimated in this case. The larger number of free parameters in approach i than in approach iv (27 versus 11, respectively) does not, however, lead to higher values of the Akaike Information Criterion (AIC); approach i (i.e., “year-by-year” and assuming equilibrium) has consistently lower AIC values.

**Fig 3:**
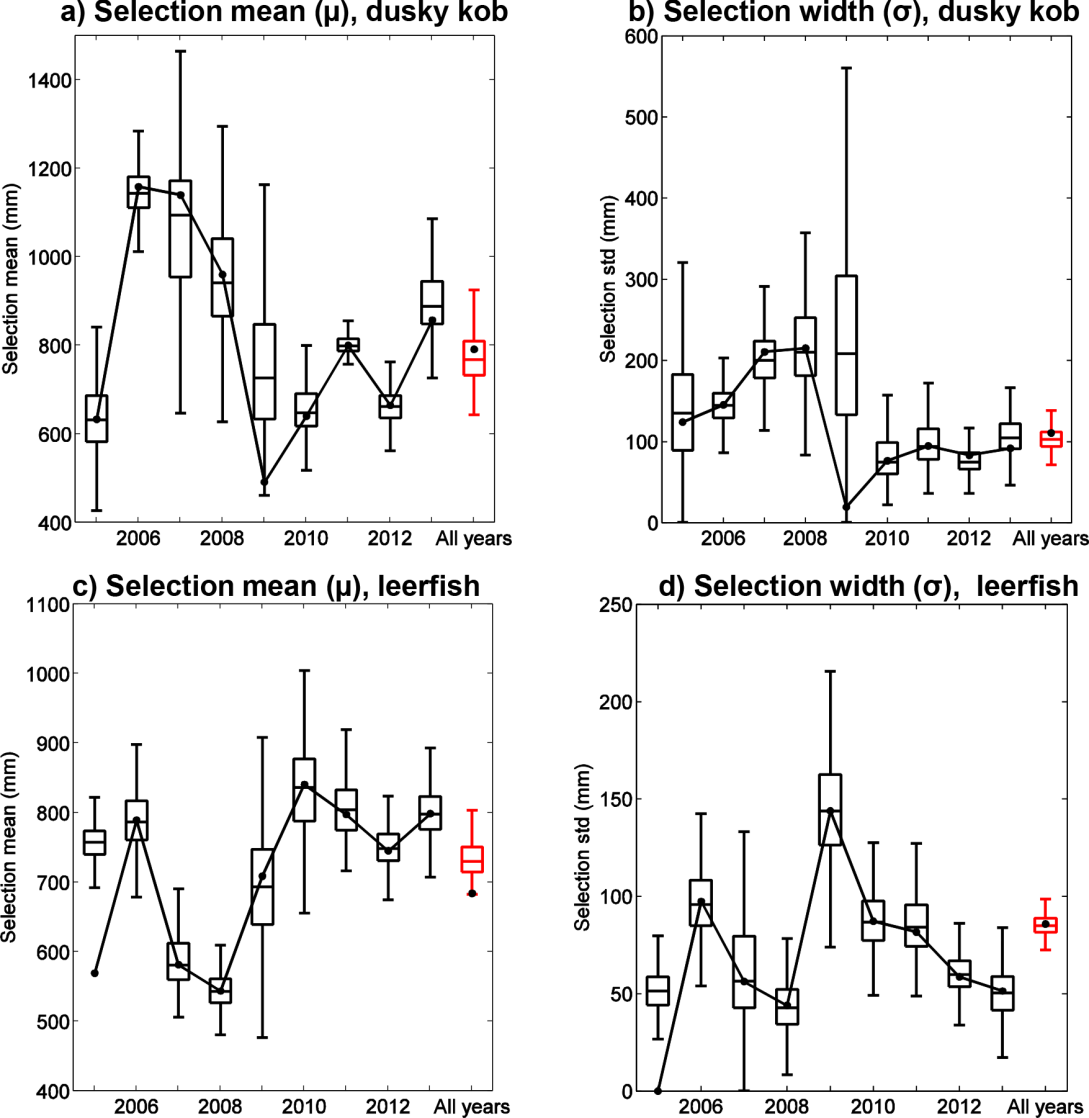
Estimation of selectivity parameters based on 2 parameter estimation approaches. Estimates of the inflection point and width of the selectivity ogive, μ_S_ and σ_S_, respectively, over 9 years for dusky kob (*A*. *coronus*) (a,b) and leerfish (*L*. *amia*) (c,d). Lines show model predictions using the original dataset, whereas box plots display distributions computed from 1000 random resamplings with replacement of individual fish lengths. Box plots show the median (center line), interquartile range (boxes), and limits beyond which data is considered to be an outlier (i.e., ~3 standard deviations for normally distributed data; whiskers). Results for two different parameter estimation approaches are presented in each panel: for approach iii, nine yearly boxplots are given, starting with 2005 to the left of each panel; and for approach iv, a single boxplot is given to the right of each panel.

Estimated fishing mortality trends differ considerably between “all years” (approaches ii and iv) and “year-by-year” approaches (approaches i and iii) (red and black curves, respectively, in Fig 2). However, these differences do not necessarily correspond to significant changes in total mortality because they may be counteracted by differences in estimated gear selectivity parameters. For example, μ_S_ from “year-by-year” calculations for dusky kob (black boxes and curve in Fig 3A) increases and decreases with respect to that from “all years” (red box in Fig 3A) in step with variability in fishing mortality estimates, with μ_S_ being higher than the value estimated for “all years” during years when the corresponding fishing mortality is higher, and vice-versa (e.g., compare Fig 2A with Fig 3A). Increasing μ_S_ (for F fixed) increases the age at which fish enter the fishery and, therefore, reduces total fishing mortality.

Differences in fishing mortality between “n-fishing-years” (approaches iii and iv) and equilibrium approaches (approaches i and ii) (solid and dashed curves, respectively, in Fig 2) are smaller than those between “all years” (approaches ii and iv) and “year-by-year” (approaches i and iii) assumptions. Nevertheless, differences are globally consistent, with higher estimated fishing mortality rates for the “n-fishing-years” hypothesis than for the equilibrium assumption. The exception to this general result is for dusky kob estimates using a single set of selectivity parameters (red curves in Fig 2A), for which fishing mortality is higher for the latter part of the time series if equilibrium is assumed than if the “n-fishing-years” hypothesis is assumed. Nevertheless, these fishing mortality rates are associated with values for selectivity parameter μ_S_ that are considerably lower for the “n-fishing-years” hypothesis than for equilibrium (792 vs 808 mm TL, respectively; Table 3), and, therefore estimated total fishing mortality may still be higher for “n-fishing-years”.

**Fig 4:**
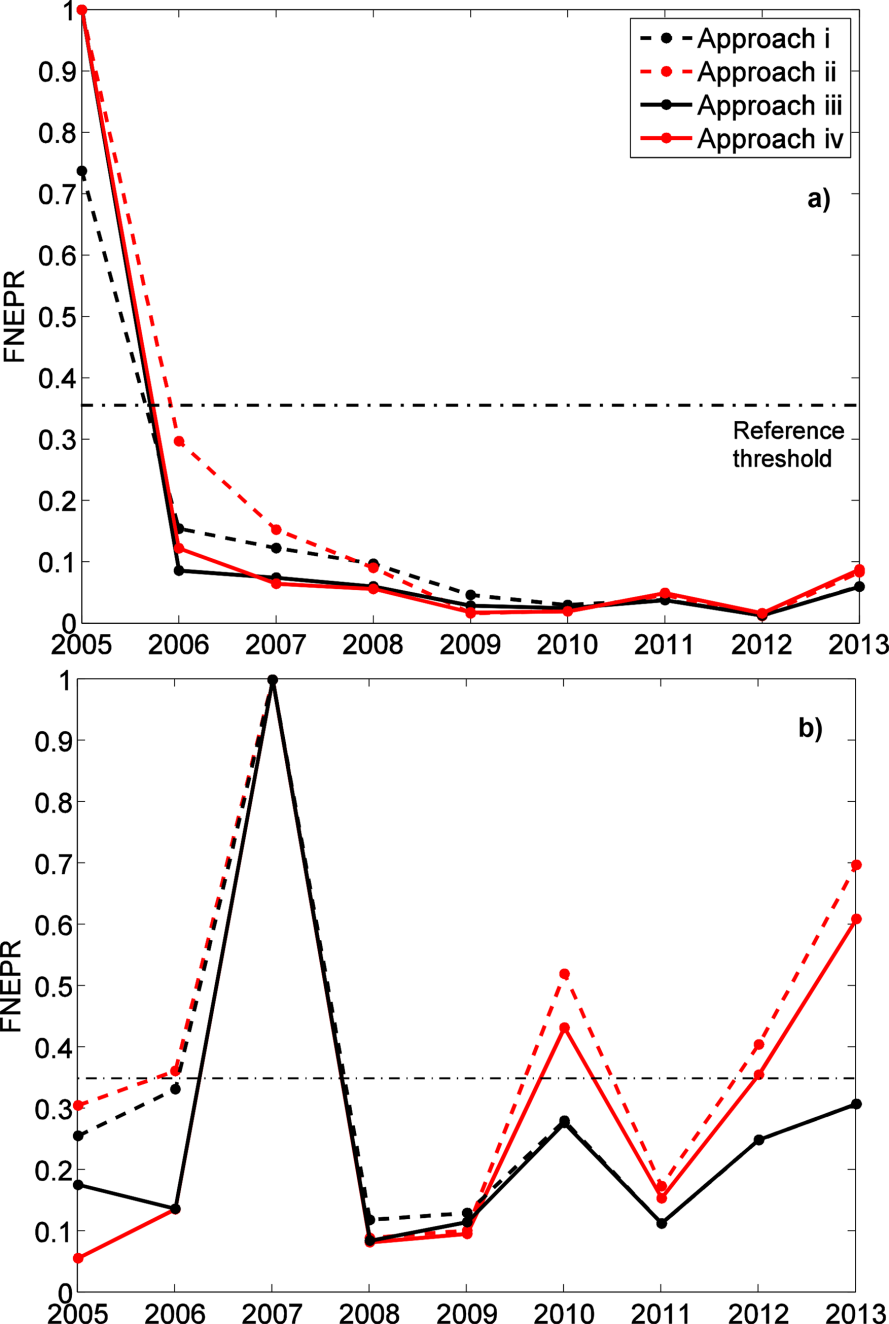
Estimation of FNEPR rates based on the 4 parameter estimation approaches. Results for dusky kob (*A*. *coronus*) and leerfish (*L*.*amia*) are presented in panels (a) and (b), respectively. Results assuming constant selectivity (μ_S_ and σ_S_ fixed over years) are presented in red (approaches ii and iv), whereas those assuming yearly selectivity parameters are in black (approaches i and iii). Dashed lines represent rates assuming the population is at equilibrium (approaches i and ii), whereas solid lines use the “n-fishing years” hypothesis (approaches iii and iv). The vertical dotted line represents an often-used limit reference point for medium to long-lived species established by Clark (2002).

Estimates of dusky kob FNEPR assuming equilibrium decline steadily and considerably since 2005 (Fig 4A), suggesting that EPR has been significantly reduced by fishing. Already in 2006, our model estimates that only 30% of the per recruit reproductive capacity with no fishing remained. FNEPR estimates after 2005 are all well below 0.35, an often-used limit reference point for management of medium to long-lived exploited fish populations [12]. Even if one disregards the zero fishing mortality estimate of 2005, the 2013 FNEPR estimate (= 0.08) is only 26% of that for 2006.

Estimates of the impact of fishing on leerfish reproductive capacity are more difficult to interpret. Though FNEPR estimates for several years, including 2005, are below the 0.35 reference point, inter-annual variability is considerable and several years have estimates above that of 2005 (Fig 4B). For 2013, the FNEPR estimate for leerfish is 0.70, more than twice the 2005 estimate. For both species, FNEPR estimate uncertainties are less notable than uncertainties for fishing mortality.

FNEPR results are generally consistent across different assumptions regarding time since the onset of fishing and variability in selectivity between years (Fig 4). Assuming equilibrium (approaches i and ii) generally leads to slightly higher FNEPR estimates than if the “n-fishing-years” hypothesis is used (approaches iii and iv), with differences between the two particularly significant at the beginning of the time series. The exception to this general rule is dusky kob for 2005 using yearly selectivity parameters, for which FNEPR is 0.7 for equilibrium (approach i) and 1 for “n-fishing years” (approach ii). However, closer examination of these results reveals that they are due to very minor differences in catch length-frequency distributions (see Fig D in S2 File) and KL divergences that are below the sensitivity of model results to life-history parameters (see the following section on Evaluation of model performance). After 2006, only 12% of dusky kob’s per recruit reproductive capacity in the absence of fishing remained based on parameter estimation approach iv and FNEPR values are thereafter approximately constant, whereas assuming equilibrium conditions (approach ii), reproductive capacity declines consistently from 2005 to 2009 (Fig 4A, solid red curve and solid black curve, respectively). Assuming “year-by-year” selectivity parameters (approaches i and iii in Fig 4A) does not significantly alter FNEPR estimates, which is coherent with previous observations that changes in selectivity were accompanied by similar changes in fishing mortality, each having an opposite effect on total fishing mortality in the population.

**Fig 5:**
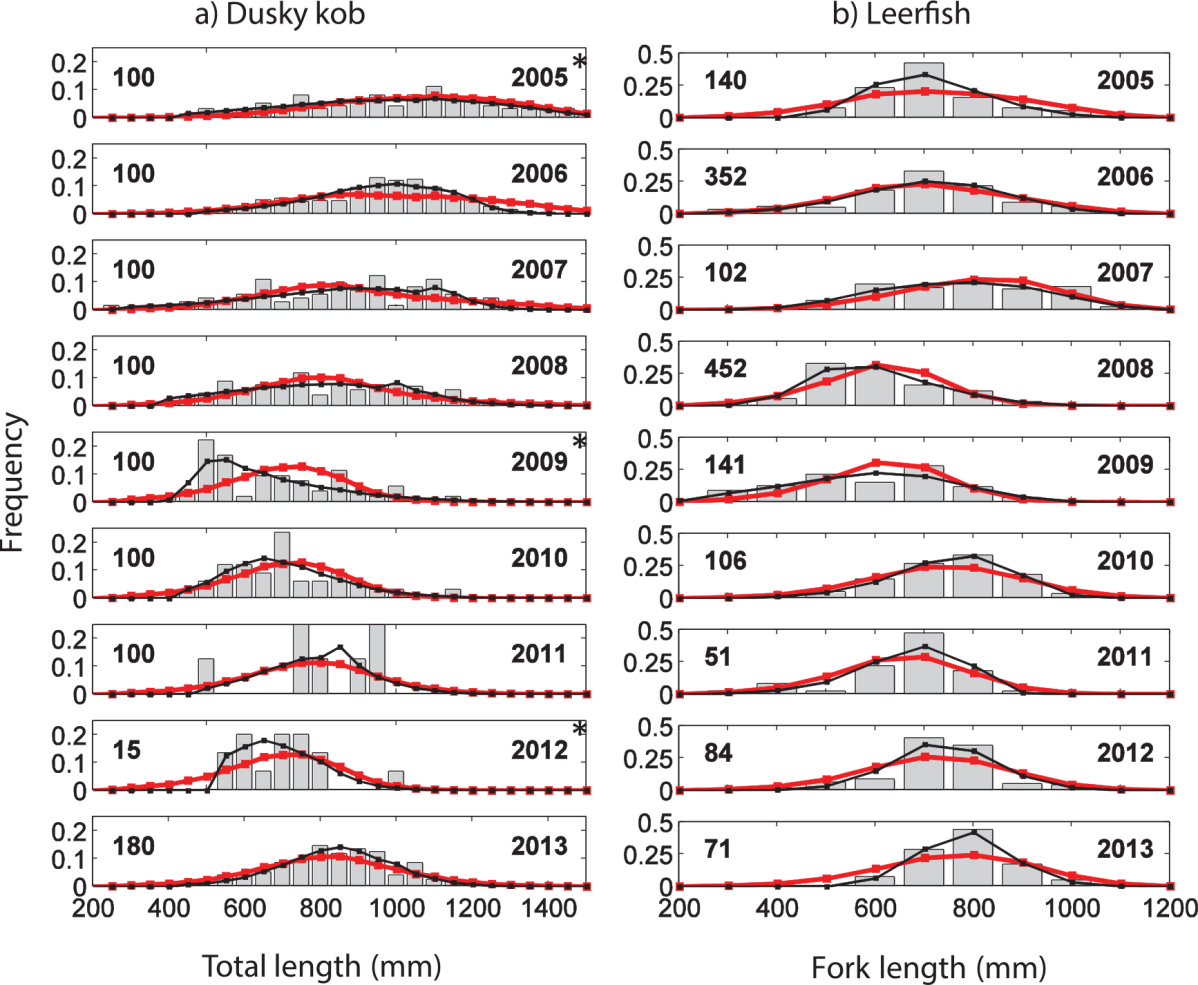
Length-frequencies and model fits. Length-frequencies from the recreational catch for dusky kob (*A*. *coronus*) (a) and leerfish (*L*. *amia*) (b) in southern Angola from 2005 to 2013. Bars are size-structured data (32 catch size classes separated into 50 mm for the dusky kob; and 11 catch size classes separated into 100 mm for the leerfish), black lines are model fits using parameter estimation approach i (“year-by-year” and equilibrium condition) and red lines are for parameter estimation approach iv (“All years” and “n-fishing years”). Number of caught fishes per year is given to the left side of the plot. Stars represent years for which the fishing mortality estimations were particularly uncertain.

For both species, model estimations of catch length-frequency distributions were able to reproduce general patterns observed in the data, such as the range of sizes captured and approximate modal catch size (Fig 5). The model, however, doesn’t reproduce multiple or secondary peaks observed in some years for dusky kob, and large peaks in either species posed problems for all estimation methods. Not surprisingly, for some years using yearly selectivity parameters produces a much closer match between observations and model predictions than when using a single set of selectivity parameters (e.g., comparing black and red curves, respectively, in Fig 5A for the year 2009). Finally, the small number of size classes used for leerfish clearly produces smoother, and perhaps less informative, length-frequency distributions than those for dusky kob.

### Evaluation of model performance

Model performance was explored using sensitivity analysis, classical explorations of likelihood surfaces and simulation experiments. We focus primarily on dusky kob because results for the two species are generally similar and dusky kob has more coherent trends in FNEPR.

**Fig 6:**
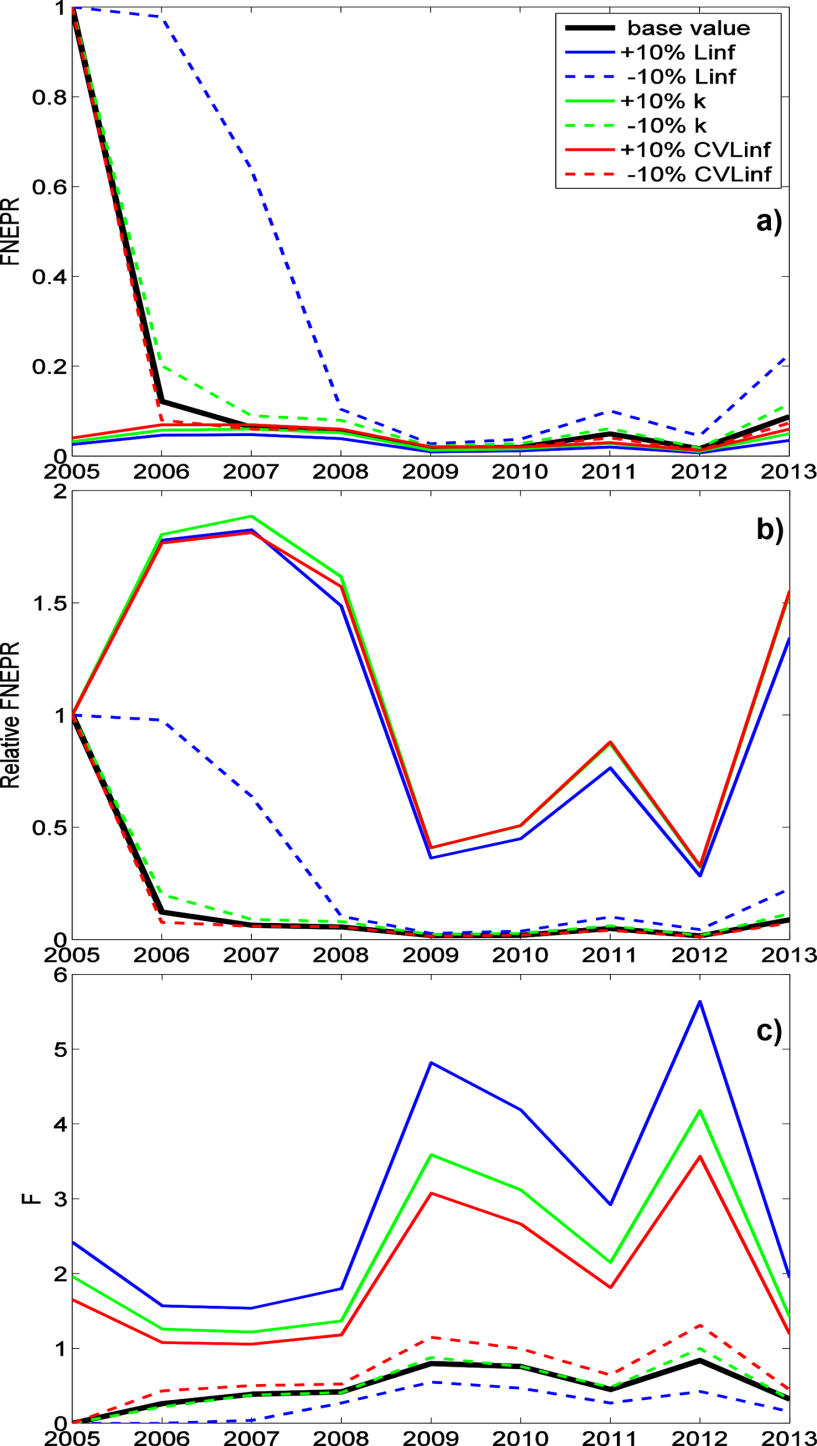
Estimation of FNEPR and F for ±10% change in biological parameters using parameter estimation approach iv. FNEPR (a), FNEPR relative to FNEPR in 2005 (b) and F (c) for dusky kob (*A*. *coronus*) when the asymptotic Von Bertalanffy length parameter (L_∞_) (blue), the growth parameter (k) (green) and the coefficient of variation of length (CV L_∞_) (red) of generated data are 10% greater (solid lines) and 10% less (dashed lines) than the normal input parameters of the estimation model. Changes in FNEPR estimates due to ±10% changes in t_0_ and M are not shown, but are similar in magnitude to those for ±10% changes in CV L_∞_). Results are shown for approach iv (“all years–n-fishing years”).

**Fig 7:**
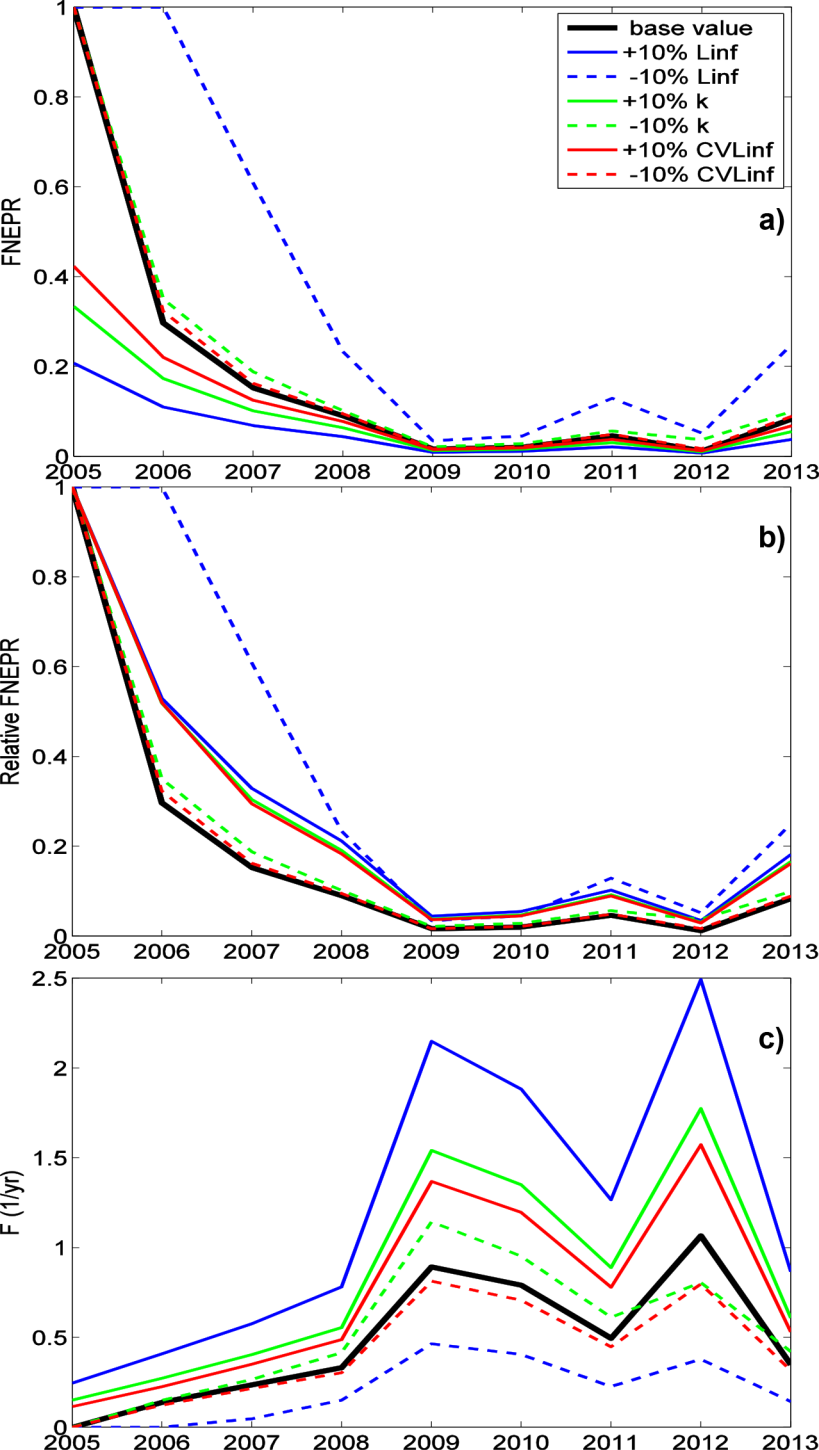
Estimation of FNEPR and F for ±10% change in biological parameters using parameter estimation approach ii. FNEPR (a), FNEPR relative to FNEPR in 2005 (b) and F (c) for dusky kob (*A*. *coronus*) when the asymptotic Von Bertalanffy length parameter (L_∞_) (blue), the growth parameter (k) (green) and the coefficient of variation of length (CV L_∞_) (red) of generated data are 10% greater (solid lines) and 10% less (dashed lines) than the normal input parameters of the estimation model. Changes in FNEPR estimates due to ±10% changes in t_0_ and M are not shown, but are similar in magnitude to those for ±10% changes in CV L_∞_). Results are shown for approach ii (“all years–equilibrium”).

The sensitivity analysis revealed that FNEPR estimates for dusky kob were particularly sensitive to (listed in approximate order of importance): the asymptotic Von Bertalanffy length L_∞_, the growth parameter k, age at zero length t_0_, natural mortality M and the coefficient of variation of asymptotic length CV L_∞_ (Table 4). An increase in any of these parameters except natural mortality lowered FNEPR estimates, whereas increasing natural mortality increases FNEPR estimates. Sensitivity was largest for the first 3 years of data (2005–2007), with FNEPR varying between 1 and ~0.1 (Figs 6 & 7). Nevertheless, when FNEPR changes are measured relative to the first year of data (as in O’Farrell & Botsford [13]), variability is considerably reduced if the data is treated as if they represent equilibrium conditions (approach ii; Fig 7B), though this is not the case for the “n-fishing-years” approach (approach iv; Fig 6B). As found in Hordyk *et al* (2015) [20], FNEPR estimates are less sensitive to input parameter values than are estimates of fishing mortality F (Figs 6C & 7C) due to the non-linear relationship between these two.

**Table 4:** Sensitivity analysis of biological input parameters on FNEPR estimates for dusky kob (*A*. *coronus*). Results are shown for the approach ii. (Equilibrium) but those for other modeling approaches are similar.

Years	2005	2006	2007	2008	2009	2010	2011	2012	2013
L_∞_	-7.93	-7.03	-4.56	-1.43	-0.18	-0.25	-0.83	-0.40	-1.66
k	-9.51	-1.77	-0.73	-0.38	-0.05	-0.08	-0.21	-0.23	-0.40
t_0_	-5.99	-0.90	-0.32	-0.15	-0.01	-0.01	-0.09	-0.02	-0.17
CV L_∞_	-5.77	-0.77	-0.28	-0.13	-0.01	-0.01	-0.08	-0.05	-0.15
M	5.78	0.79	0.30	0.15	0.01	0.03	0.09	0.04	0.17
b	0.00	-0.09	-0.08	-0.06	-0.02	-0.03	-0.05	-0.02	-0.06
μ_mat_	0.00	-0.22	-0.20	-0.17	-0.08	-0.09	-0.13	-0.06	-0.17
σ_mat_	0.00	0.01	0.02	0.02	0.02	0.02	0.02	0.01	0.02

**Fig 8:**
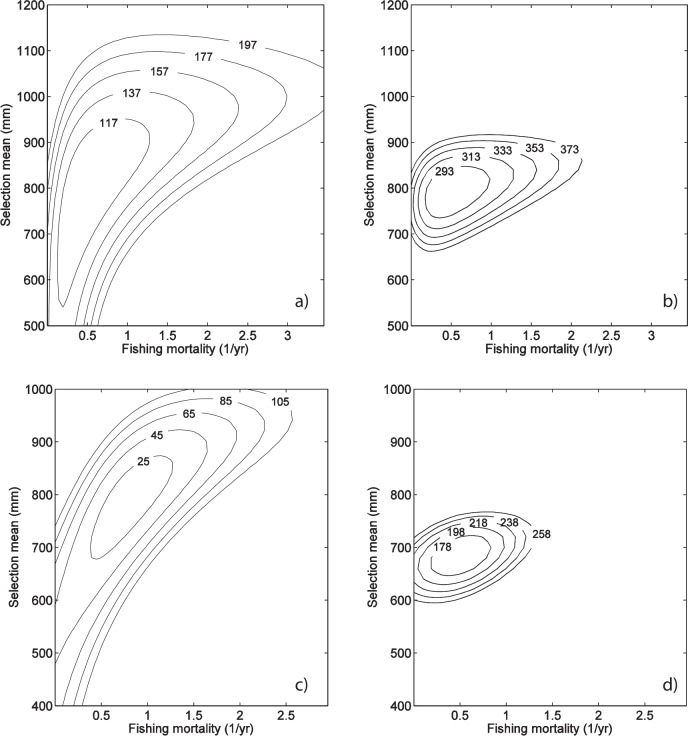
Likelihood surface profiles. Exploration of KL divergence values as a function of the value of the inflection point of the selectivity ogive μ_S_ and the fishing mortality F calculated for dusky kob (*A*. *coronus*) in 2011 (a, b) and for leerfish (*L*. *amia*) in 2006 (c, d). In all panels, fishing activity is assumed to start in 2005 (i.e., the “n-fishing-years” hypothesis is used). Results for parameter estimation approaches iii and iv are shown: year-by-year selectivity (a, c) and constant selectivity (i.e., “all-years”; b, d). For both years, 100 fishes were caught of the respective species.

Parameter identifiability in our approach was qualitatively assessed by comparing plots of KL divergence surfaces as a function of F and μ_S_ (all other parameter values fixed at their optimal values) between different approaches for estimating selectivity parameters (“year-by-year” vs “all years”). For the “year-by-year” estimation approach (approach iii), contours of the divergence surface around the minimum value were elongated and diagonal (Fig 8A and 8C), indicating problems of identifiability and linked uncertainties in F and μ_S_ [13]. When selectivity was maintained constant over all years (approach iv; Fig 8B and 8D), the contours of the KL divergence surface around the minimum values were rounded ellipsoids and uncertainties in F and μ_S_ were reduced and largely uncorrelated. These observations were confirmed by the number of identifiable parameters based on counting the number of eigenvalues of the hessian matrix of the likelihood function superior to a threshold of 0.01 following the approach of Reboulet *et al*. [42]. Using this approach, 9 parameters were found to be identifiable for both the “all years” and “year-by-year” approaches, despite these methods having 11 and 27 estimated parameters, respectively.

**Fig 9:**
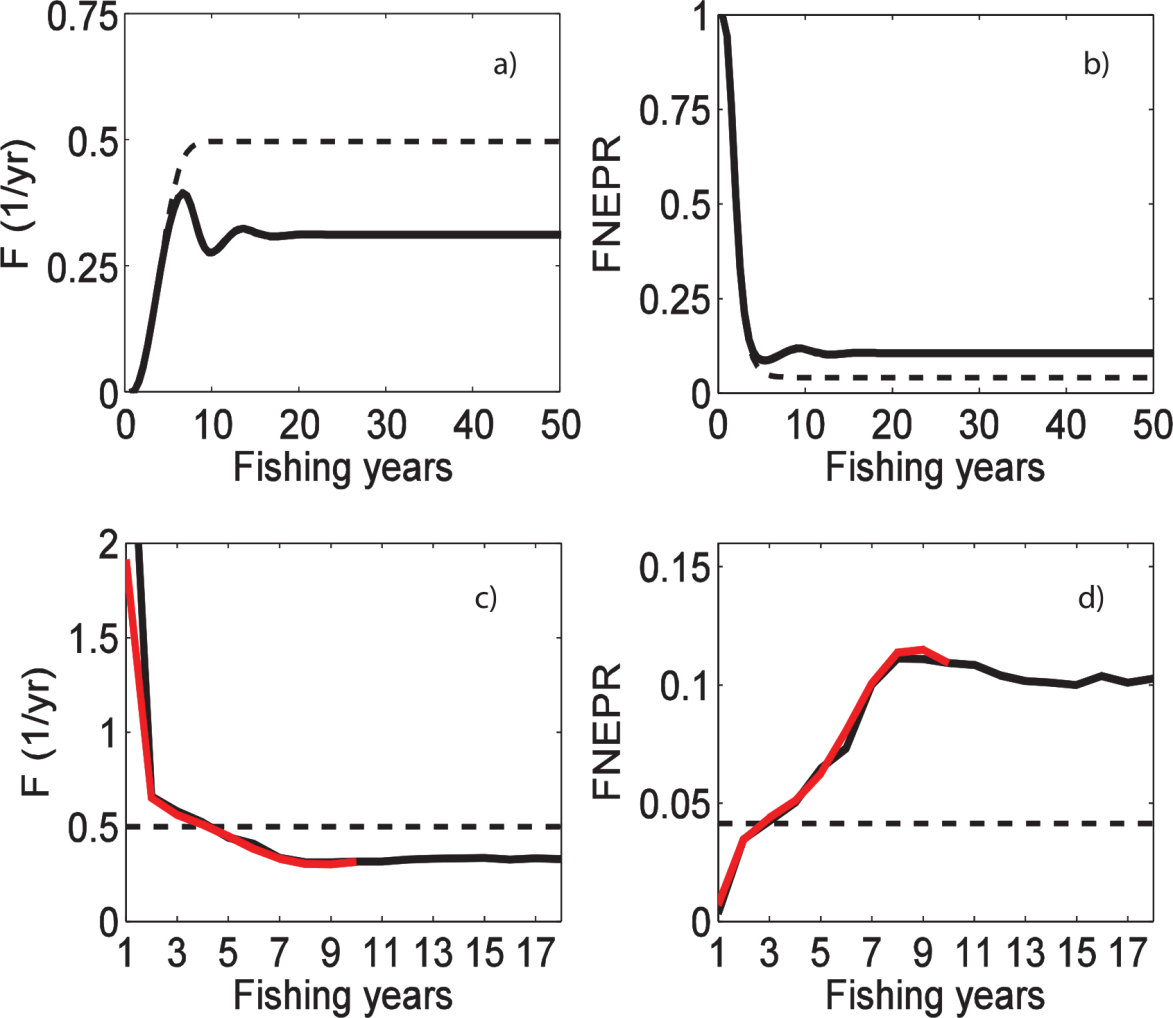
Fishing mortality and FNEPR from simulations 1 and 2. Fishing mortality rates (a, c) and FNEPR (b, d) estimates for dusky kob (*A*. *coronus*) from simulated size distributions. Results assuming year-by-year selectivity parameters, i.e. from approach i (a, b) are presented for a period of 100 years for constant recruitment (i.e., simulation 1; dashed lines) and recruitment limitation (i.e., simulation 2; solid lines). Results from parameter estimation approach iv (c, d) and simulation 2 are presented for 9 years (as in our real data; red lines in c, d) and 18 years (black lines in c, d) of simulated catch length-frequency distributions. Fishing mortality and FNEPR values for constant recruitment are respectively 0.5 yr^-1^ and 0.041% (dashed lines).

“Transient phase” and “recruitment limitation” simulations were used to understand the robustness of our approach to non-equilibrium conditions. Parameter estimation approach i underestimated fishing mortality and overestimated long-term FNEPR values during the initial years (5–10 years; approximately 0.5–1 generations) after the onset of fishing (Fig 9A and 9B; Table 5). If recruitment was held constant (as in simulation 1), fishing mortality and FNEPR estimates stabilize on the correct values of 0.5 yr^-1^ and 0.041, respectively, after one generation. When we allowed for recruitment limitation (as in simulation 2), fishing mortality estimates oscillated before stabilizing on a value of approximately 0.3 yr^-1^, after approximately two generations. This was considerably lower than the input fixed value of 0.5 yr^-1^ (solid curve in Fig 9A). FNEPR estimates were correspondingly higher than the expected value (solid curve in Fig 9B). This biased estimation continued no matter how long the simulation was continued. Precision of parameter estimates improved in parameter estimation approach iv during the initial years after the onset of fishing, but this approach did not remove the persistent, long-term bias in estimations when the population was allowed to collapse (Fig 9C and 9D). If recruitment was constant (dashed horizontal line in Fig 9C and 9D), the “n-fishing years” method exactly reproduced the expected values of fishing mortality and reproductive capacity for all years. For a collapsing population (solid curves in Fig 9C and 9D), initial mortality estimates exceed the expected value, but estimates over longer time periods approached those of parameter estimation approach i, underestimating F and overestimating FNEPR.

**Table 5:** Parameter estimates from simulated size distributions for different simulations for dusky kob (*A*. *coronus*). Values in the first line shown in bold indicate the “true” values used to generate simulated data. Selectivity parameters (inflection point of the selectivity ogive μ_S_ and width of the selectivity logistic curve σ_S_) are expressed in mm total length.

Model output	μ_S_ (mm)	σ_S_ (mm)	F (yr^-1^)	F (yr^-1^)	FNEPR	FNEPR
Year			1	9	1	9
*"True" values*	**792**	**111**	**0.50**	**0.50**	**0.041**	**0.041**
*1*. *Transient phase*						
i. Year by year—equilibrium	780 ^a^	109 ^a^	3.06e^-6^	0.4989	1.00	0.041
iii. All years—equilibrium	769	109	0.0024	0.46	0.97	0.042
iv. All years—n fishing years	792	111	0.50	0.50	0.041	0.041
*2*. *Recruitment limitation*						
i. Year by year—equilibrium	811 ^a^	94 ^a^	3.04e^-6^	0.31 ^b^	1.00	0.11 ^b^
iv. All years—n fishing years	828	96	1.91	0.31	0.0070	0.11
*3*.*4*. *Stochasticity*						
cv R^c^ = 0.2 ^d^	792	111	0.49	0.50	0.044	0.041
Small sample size ^d^	798	111	0.51	0.51	0.043	0.041

^a^ Mean of values’ series because selectivity parameters were estimated independently for each year.

^b^ Values after 100 years.

^c^ Recruitment coefficient of variation.

^d^ Median of 1000 bootstraps.

**Fig 10:**
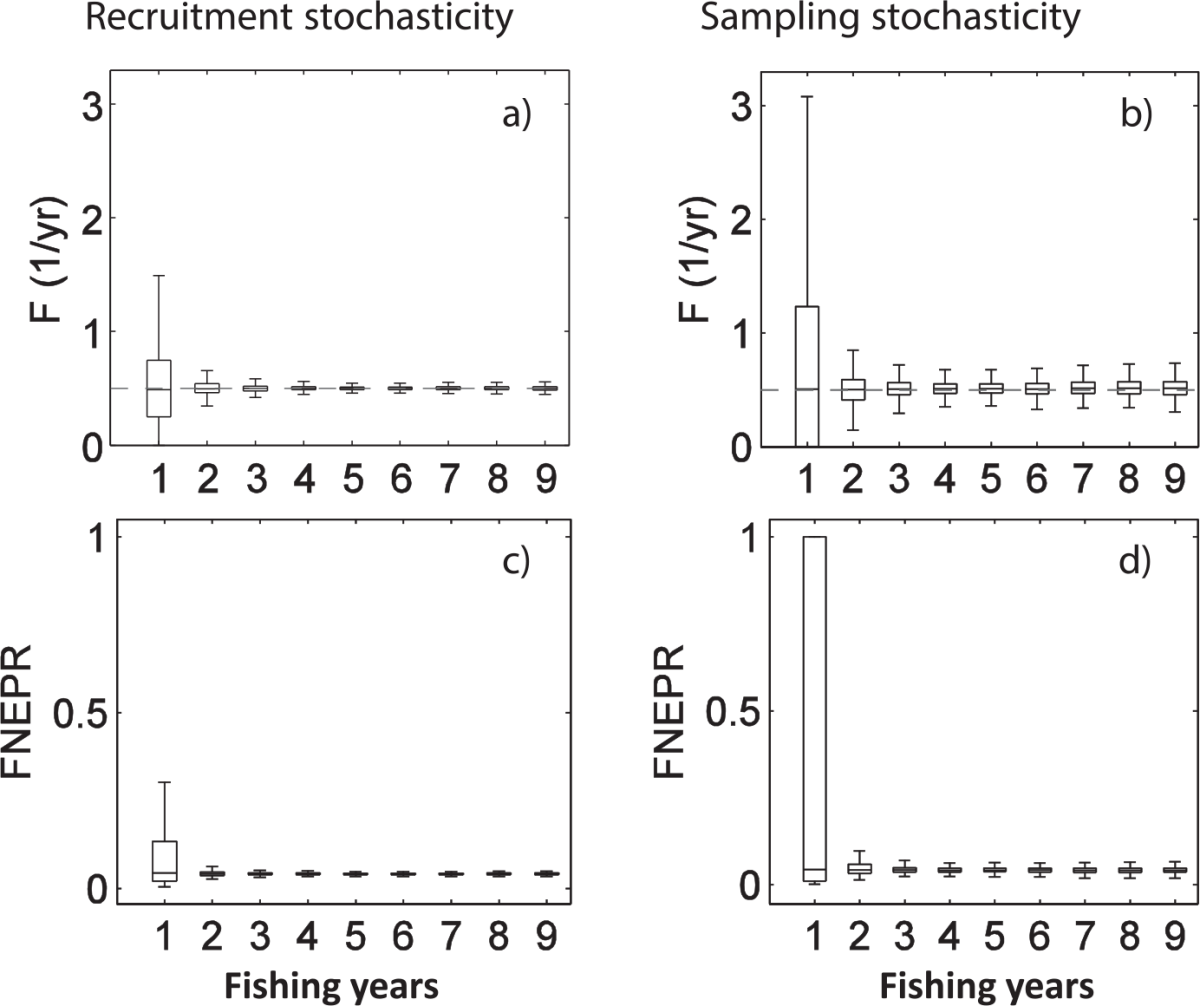
F and FNEPR uncertainty estimates from simulations 3 and 4. Uncertainty in F and FNEPR for dusky kob (*A*. *coronus*) from simulated size distributions from simulations with recruitment stochasticity (a, c; simulation 3) and sampling stochasticity (100 fishes) (b, d; simulation 4). Results for parameter estimation approach iv are shown. Box plots show the median (center line), interquartile range (boxes), and limits beyond which data is considered to be an outlier (i.e., ~3 standard deviations for normally distributed data; whiskers). Variability was similar for leerfish (see Fig G in S2 File).

In addition to the biases identified above, estimates for the first years after the onset of fishing were also subject to higher uncertainty when stochasticity was included in the simulated data (Fig 10). Both recruitment and sample stochasticity simulations (i.e. simulations 3 and 4) had similar qualitative effects on uncertainty in fishing mortality and FNEPR estimates, producing considerable estimation uncertainty for the first years after fishing began, but having relatively small impacts on the results after 2–3 years. Apart from the first year after fishing began, uncertainties in parameter estimates from simulations with sample stochasticity were inferior to uncertainty estimates from bootstrapping over real data (e.g., compare Fig 10 with Figs Ca and Da in S2 File).

## Discussion

### Methodological changes

One of the objective of this study was to improve on an existing method for estimating harvest characteristics and population status from length-frequency data [13]. A number of individually-small methodological changes were made to the original approach of O’Farrell & Botsford [13] and collectively, they added flexibility and robustness to the approach.

Given the previously identified covariance between fishing mortality and selectivity parameter estimates, the impact of assuming constant selectivity patterns over the study period was investigated. The assumption of constant selectivity is reasonable if there is no evidence of changing fishing practice over time. A single set of selectivity parameters considerably reduced the number of estimated parameters, producing a model that has relatively few problems of identifiability. Contours of the KL divergence surface around the minimum values were tighter and more closely aligned with parameter axes when a single set of selectivity parameters was used, indicating reduced uncertainty and lack of correlation between parameter values for F and μ_S_.

Surprisingly, this reduction in the number of free parameters did not produce smaller values of AIC than the original model using a year-by-year selectivity assumption. This result suggests that, though there is considerable uncertainty regarding annual selectivity parameters, selectivity is changing significantly over time. Given the technology creep in recreational fishing tackle and the range of tackle used by fishers in this study, variability in selectivity among years is indeed possible. Selectivity changes over time could also be driven by non-equilibrium dynamics due to spatial and temporal variation of fishing effort combined with movement of different subpopulations in and out of the study area [43]. Another possible source of temporal variability in selectivity is the variety of fisheries targeting the study species. In our approach, we implicitly assumed selectivity of the recreational fishery is not significantly different from that of the average selectivity applied to the stock by all fisheries. Whereas selectivity of the foreign recreational fishery data used in this study is likely reasonably close to that of the local recreational fishery and the inshore artisanal line fishery, it is potentially quite different from that of the illegal inshore gillnet and beach-seine fisheries. Unfortunately, without fishery-specific catch and catch length-frequency data, it is difficult to assess the impact of this assumption on our results. In principle, this problem could be addressed by having separate selectivity parameters for extractive fisheries and the catch-and-release observation process. This would be an interesting avenue for future research if fishery-specific data can be collected to validate results.

We have also integrated some aspects of the temporal evolution of fishing pressure into the method, namely the ability to assume that fishing began at some finite time in the past. This is roughly correct for the southern Angolan recreational species that were lightly exploited before approximately 2005. Estimations with “n-fishing years” identified more quickly than other parameter estimation approaches trends in fishing pressure likely to cause long-term overexploitation. During the initial years of the time series, the equilibrium assumption produced lower estimates of total fishing mortality, and consequently higher estimates of FNEPR, than those for the “n-fishing years” hypothesis. This is because fishing over many years requires a lower annualized rate of fishing mortality to produce a given change in size structure than fishing over a short time period. One disadvantage of using the “n-fishing years” approach was that it lead to high estimates of fishing mortality from the beginning of the fishery, and therefore comparisons between early and late estimates of FNEPR [13] are less valuable. This suggests that applying both approaches to a given dataset can provide valuable complementary information regarding real exploitation levels (“n-fishing-years”) and long-term changes in population persistence (equilibrium assumption).

We also used simulations to examine possible sources of bias and uncertainty in our methodology due to small sample size and the underlying assumption of constant recruitment. When we integrated recruitment limitation, as expected in an overexploited population, the method predicted (several years after the start of the fishery) a lower fishing mortality rate, and correspondingly higher FNEPR, than was actually occurring. This result is undesirable and quite problematic if not recognized because it could lead to overly optimistic status evaluations [44]. The explanation for this result is related to how fishing changes the age and size structure of fish populations. The most obvious impact of fishing is that length-frequency distributions are right-skewed. Less obvious is that recruitment collapse due to overfishing can also skew length-frequency distributions to the left. As recruitment decreases, fewer young individuals enter the population, increasing the relative abundance of old to young individuals. The combination of these two competing processes results in mortality estimates that are elevated, but are lower than is actually occurring.

Paradoxically, during the initial years of the fishery, the “n-fishing-years” methodology using a single set of selectivity parameters overestimated mortality rates. This is because initially recruitment was not limited despite overfishing. As we estimated only one selectivity curve across all years, underestimation of fishing rate in later years forced the method to overestimate the fishing rate during the early stages of fishing.

Recruitment and/or sample stochasticity both had similar effects on exploitation estimations. Similar to what was previously observed by O’Farrell & Botsford [13] for California rockfish using their equilibrium-based approach, uncertainty in estimates for dusky kob and leerfish is high during the early stages of fishing before dropping to consistently low levels. After the initial years, the population size-structure represents an average over multiple years of exploitation, and therefore uncertainty levels due to stochasticity decrease. In our simulations, the levels of uncertainty (excluding the first couple of years of fishing) were below observed annual uncertainty levels in parameter estimates (compare Fig 10 with Figs Ca and Da in S2 File). This indicates that the levels and types of stochasticity examined in our simulations were not sufficient to explain observed variability on their own. Either the levels of stochasticity were higher than those explored in our simulations or other sources of uncertainty, such as variability in selectivity parameters (discussed above) or bias in biological parameter estimates, may have influenced the results. Our sensitivity analysis of the impact of baseline biological parameters values on FNEPR estimates indicates that growth parameter values have a large impact on results, and, therefore, improving the accuracy of these parameter estimates will reduce model uncertainty.

The current model can be improved in several ways. Firstly, the objective function, the Kullback-Leibler divergence, used in this study does not account for the number of observations (i.e., lengths) used. Using a classic multinomial likelihood or a Dirichlet-Multinomial would account for sample size and more naturally address issues of data over-dispersion [45]. Furthermore, penalized likelihood models and mixed effect methods are increasingly used to account for spatio-temporal variability in estimated parameters, such as recruitment, fishing mortality or selectivity [46–48]. The penalized likelihood is an objective function formed by multiplying the likelihood of parameters given the data by a penalty for random parameter variability leading to variability in population dynamics [49]. Mixed-models include both fixed and random effects, allowing many data types and model assumptions to be analyzed simultaneously in more flexible integrated analysis [47]. All of these approaches represent interesting avenues for future research, though some may ultimately be inappropriate for very limited datasets like those analyzed here.

### Study population status

Although both study species fishes were fast growing, large piscivores and exploited in the same fishery, our estimates of their exploitation status were quite different. The west coast dusky kob showed a clear long-term decline in FNEPR levels both in absolute and relative terms (Figs 4A & 7B), with the 2013 FNEPR estimate well below 1 (i.e., the value in the absence of fishing). By contrast, leerfish FNEPR estimates (Fig 4B) were higher and have considerably more inter-annual variability. Nevertheless, reaching conclusions about the exploitation status of either population requires an examination of not only the FNEPR, but also the consistency of results among years, relative trends over time and possible sources of bias or uncertainty in estimates. Here, we take these elements into account for the two study species.

#### West coast dusky kob: Population experiencing severe overfishing?

We estimate that dusky kob has experienced declines in EPR since 2005 to levels that suggest that persistence is impaired. Absolute declines have been substantial since 2005, with the 2013 EPR estimate less than 10% of the value in the absence of fishing. Perhaps more importantly, estimates show a coherent trend among years, and declines relative to either 2005 or 2006 (if one does not trust the zero fishing mortality estimate for 2005) are substantial (2013 FNEPR is approximately 26% of that for 2006). These results are consistent with the intrinsic vulnerability of large sciaenid fish to fishing [50]. Given that 88% of the fish captured at the Cunene Estuary site during this study were below the size at sexual maturity [36], it seems likely that this exploitation has contributed to recruitment overfishing in the area.

The one significant question mark regarding fishing mortality estimates for west coast dusky kob is the zero estimate in 2005. Though this could be because fishing pressure was truly very small before 2005 (when the data collection started, there was hardly any other fishing effort taking place), a more likely explanation is that inaccurate growth estimation (e.g., slightly biased towards smaller maximum size) has made it difficult for our method to estimate fishing mortality when fishing pressure is low. Exploring the sensitivity of our method to this sort of parameter bias revealed high sensitivity of egg production rates to the asymptotic length parameter. Bias in this parameter towards a small maximum size for dusky kob may be due to the limited number of large size individuals collected for estimating growth parameters (only 1.6% of the individuals were >1400 mm TL, despite an estimated L_∞_ of 1856.1 mm TL), perhaps even linked to exploitation itself prior to the study period.

An alternative interpretation (to overexploitation) of the estimated decline of the west coast dusky kob population in southern Angola is that the species has undergone a distributional shift from southern Angola into northern Namibia. Such a shift is consistent with an observed increase in northern Namibia of the density of west coast dusky kob relative to that of the closely-related, but more-southerly silver kob (*A*. *inodorus*) from 1:9 in 1993 to 4:6 in 2009 [51]. Adult west coast dusky kob appear to be sensitive to water temperatures, having evolved a seasonal southward migration in Austral summer to avoid warm (>20°C) water temperatures [51]. Given the average observed increase in sea surface temperature of approximately 0.8°C/decade in southern Angola [51,52], a distributional shift to avoid warm water intrusions from the Angola Current into southern Angola during austral winter is not impossible [36,53]. Future research could clarify the relative importance of these different mechanisms driving population trends by collecting and applying our modeling approach to catch length-frequency distributions for west coast dusky kob from northern Namibia, as well as by examining movements and physiological temperature tolerances of this species during austral winter.

#### Leerfish: Uncertain status

Rapid growth and early maturity appear to be advantageous in sustaining the leerfish population when faced with heavy fishing pressure. These traits are consistent with FNEPR estimates that are relatively high compared with those for the west coast dusky kob, but do not provide adequate explanation for large variability in FNEPR estimates between years. There are a number of possible explanations for the observed variability in leerfish estimates, three of which are considered here. Leerfish has a shorter life cycle than dusky kob (based on inverse of natural mortality rate: 1/0.28 = 3.5 years for the leerfish, whereas 1/0.12 = 8 years for the dusky kob). Moreover, the Von Bertalanffy growth parameter, k, for leerfish is three times higher than that for dusky kob, and, therefore, leerfish reaches the asymptotic Von Bertalanffy length faster. Its shorter life expectancy makes it more sensitive to recruitment fluctuations and could explain the observed instability in FNEPR estimates. Nevertheless, FNEPR uncertainty levels due to recruitment stochasticity are similar for the two species and considerably smaller than observed inter-annual variability (Fig 10). This indicates that either recruitment variability is much larger in leerfish than in west coast dusky kob (not impossible given observed CV values for recruitment considerably larger than the value of 0.2 used in simulation 3 [54]), or that recruitment variability does not entirely explain observed patterns.

Another possible explanation is that life-history parameters are not correctly estimated for leerfish. Because of its shorter life cycle, few juvenile individuals were captured during the course of the study and estimation of growth and mortality of young individuals was more difficult than for dusky kob. If, for example, this led to overestimation of leerfish growth, this could produce fishing mortality estimates that are high even if real fishing pressure is not. How poor life-history parameter estimates can account for inter-annual variability is less clear, but using nonparametric methods based on penalized-likelihoods could produce more stable parameter estimates [49]. Input parameter uncertainties could be reduced by studies specifically targeting growth of juvenile leerfish.

Finally, inter-annual variability may be caused by not sampling the same study population every year. This could be due to large-scale movement of individuals either for reproductive purposes or in response to changing environmental conditions. One hint that these factors may be important is the leerfish sex ratio. In South Africa, the sex ratio was reported to be 1M:1F [31], but in the Angolan population there are about two females to every male (1M:1.9F) [35], and females become more dominant with increasing fish size. This skewed sex ratio suggests different life histories for males and females and possible spatial segregation. Detailed recording of leerfish sexes every year and examination of otolith microchemistry to identify possible migrations may provide insight into these mechanisms. Finally, the integrated models described earlier could include sex-composition sampling to account for this sexually dimorphic growth [55] and would be an interesting avenue for future investigation.

## Conclusion

Our results indicate that the west coast dusky kob is showing signs of overexploitation, whereas the status of leerfish is less certain. Based on simulation results for collapsing populations and the fact that we have not considered the potential impact of maternal age effects [56], it is likely that the estimated declines in EPR are minimum estimates of decline relative to the unfished EPR level. According to the guidelines proposed by the FAO (2001), which use life-history traits to classify finfish species by productivity, leerfish and dusky kob fall into the low productivity category and are expected to sustain only low levels of fishing effort. Furthermore, because both of them are large piscivorous species, they are considered likely to decline faster than species further down the food chain [57]. Collectively, these factors suggest that overexploitation of these resources is likely and management action is needed.

## Supporting Information

S1 File Matlab code for length-frequency estimation model. (ZIP)

S2 File Supplementary figures. (PDF)

S3 File Supplementary tables. (PDF)
