# Supplementary material for: Data-Limited Population-Status Evaluation of Two Coastal Fishes in Southern Angola Using Recreational Catch Length-Frequency Data
Source: PLoS One. 2016 Feb 1;11(2):e0147834. doi: 10.1371/journal.pone.0147834 (PMC4734550; doi:10.1371/journal.pone.0147834)
Supplement: S2 File — (PDF) [file pone.0147834.s002.pdf]

Data-limited population-status evaluation of two coastal fishes in southern Angola using recreational catch length-frequency data. Beckensteiner, J., Kaplan, D.M., Potts, W. M., Santos, C.V., O'Farrell, M.R.

S2 File

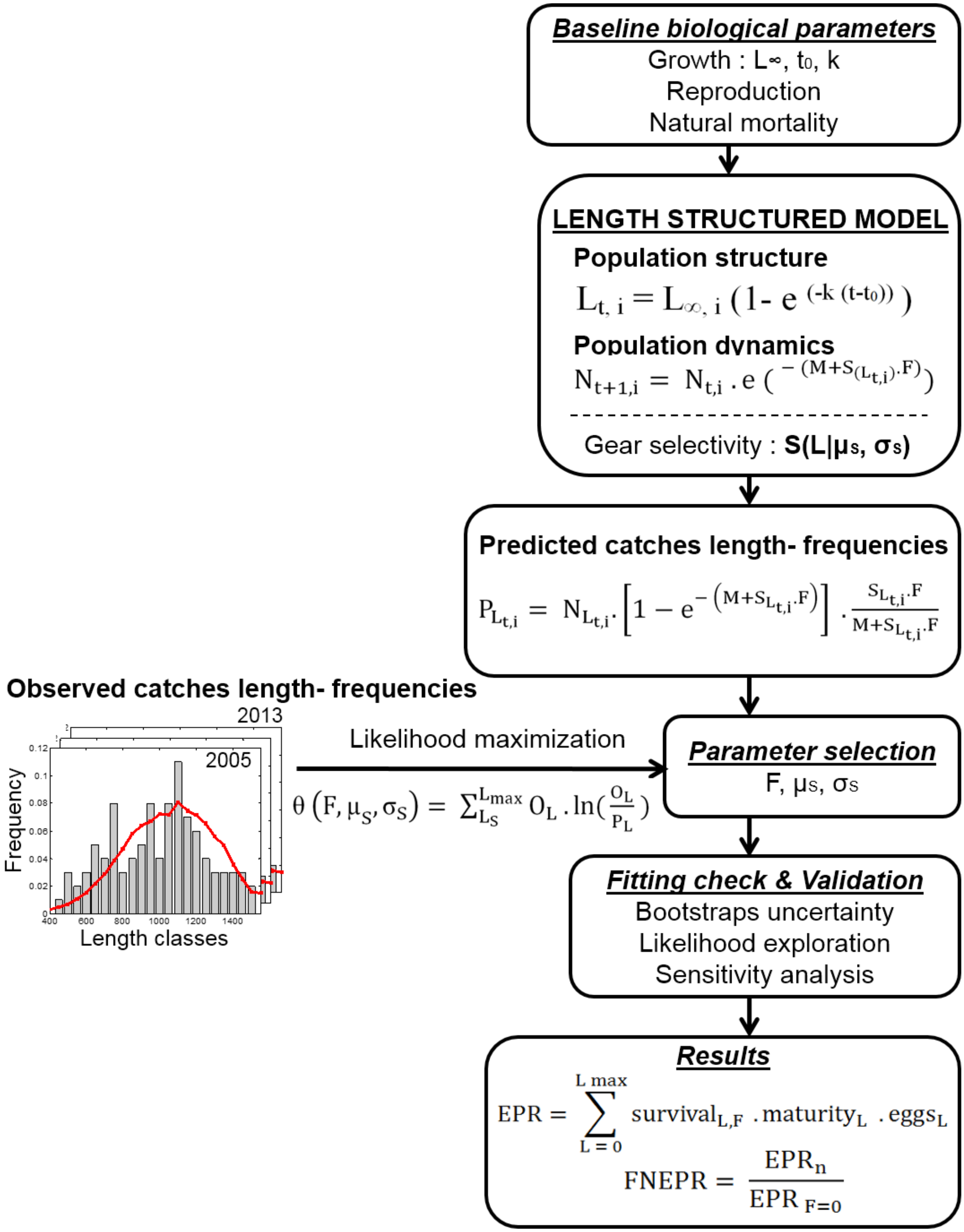

Figure A. Outline of the modeling approach used in the study.

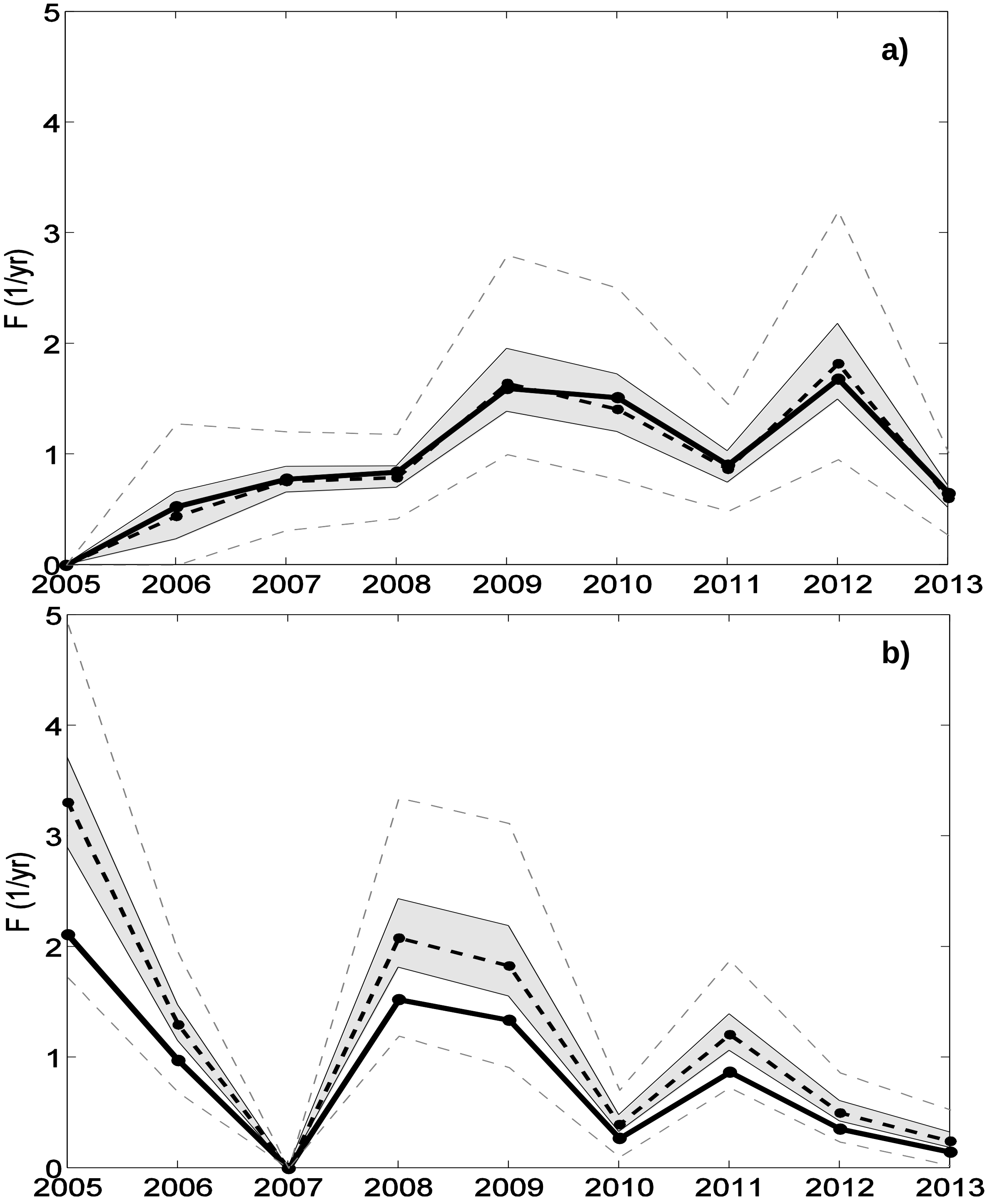

Figure B. Estimation of fishing mortality rates based on the approach iv.

Fishing mortality rates for dusky kob (*A. coronus*) (a) and leerfish (*L. amia*) (b). Intervals display fishing mortality distributions computed from 1000 random: parameter estimates recovered the median of box plots (center dashed line), grey intervals show interquartile range, and dashed lines limits beyond which data is considered to be an outlier (i.e., ~3 standard deviations for normally distributed data; whiskers).

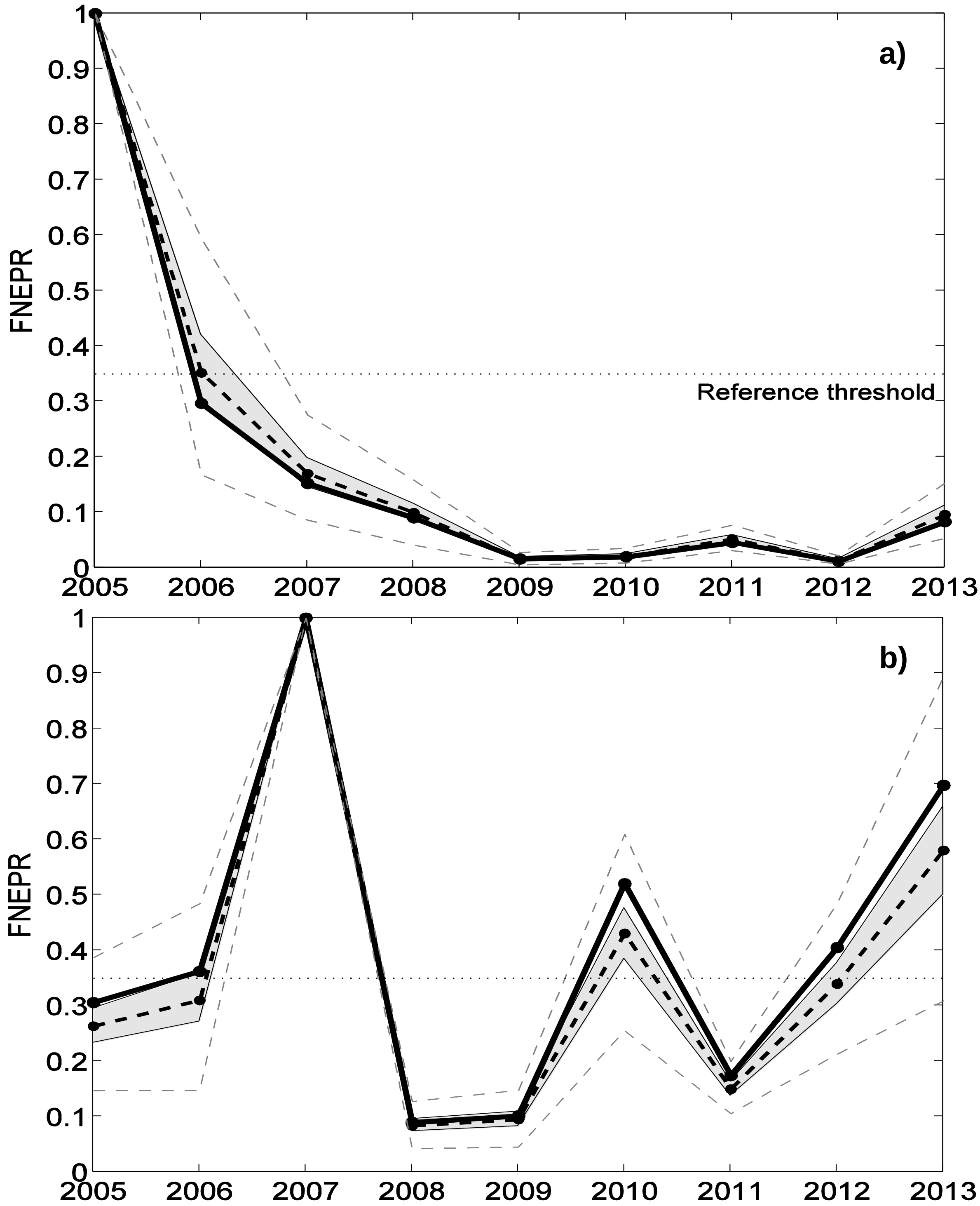

Figure C. Estimation of FNEPR rates based on the approach ii.

Fractional natural eggs per recruit rates for dusky kob (*A. coronus*) (a) and leerfish (*L.amia*) (b) assuming constant selectivity parameters at equilibrium. The dotted line represents an often-used limit reference point for medium to long-lived species established by Clark (2002).

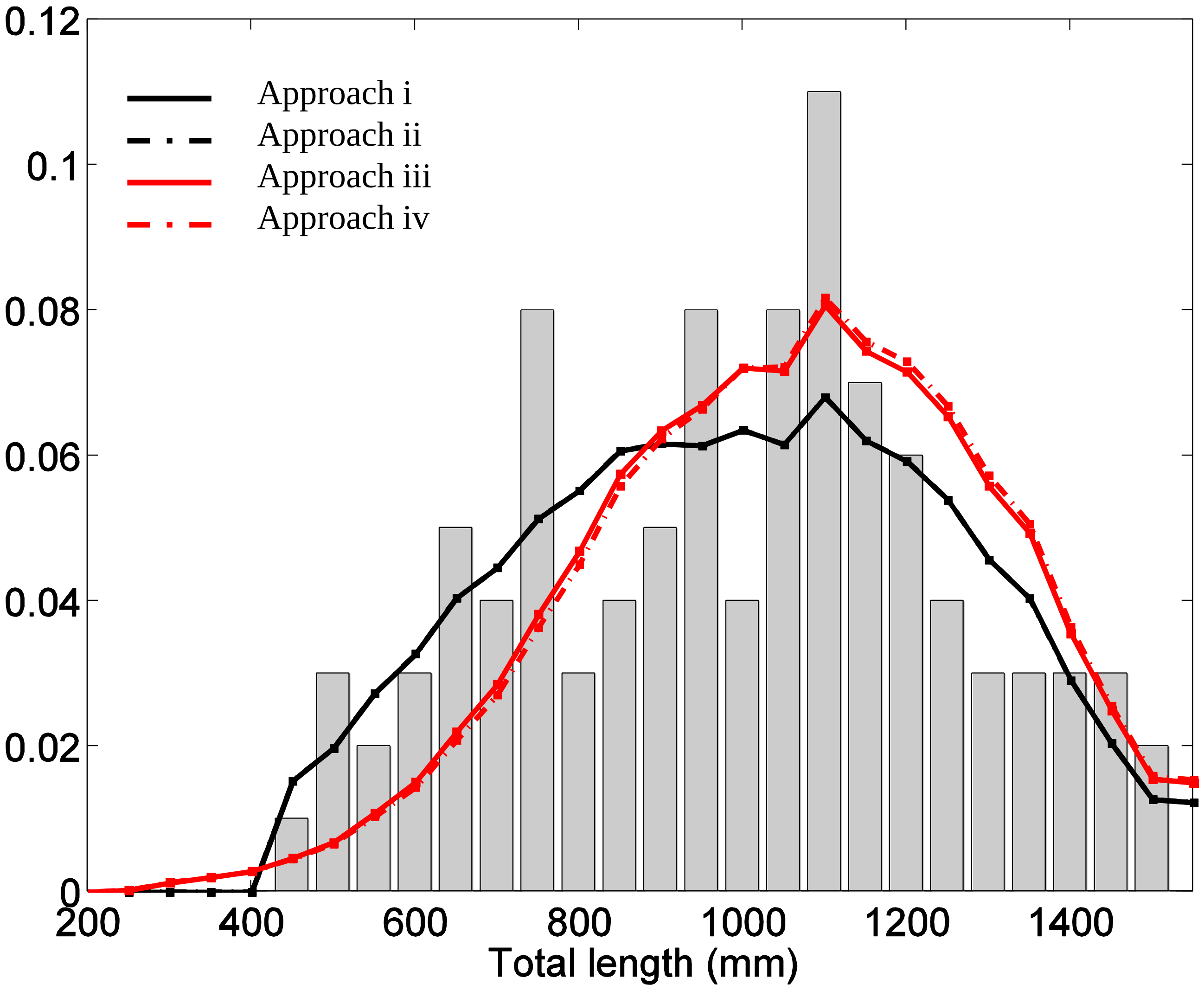

Figure D. Length-frequencies and model fits in 2005 based on the 4 parameter estimation approaches. Length-frequencies from the recreational catch for dusky kob (*A. coronus*) in 2005. Bars are size-structured data (32 catch size classes separated into 50 mm). Model fits assuming constant selectivity (μ_S and σ_S fixed over years) are presented in solid lines, whereas those assuming yearly selectivity parameters are presented in dashed lines. Black lines represent rates assuming the population is at equilibrium, whereas red lines use the "n-fishing years" hypothesis. The KL divergences for approaches i, ii, iii, iv are respectively 184.7, 258.7, 193.3, 272.4.

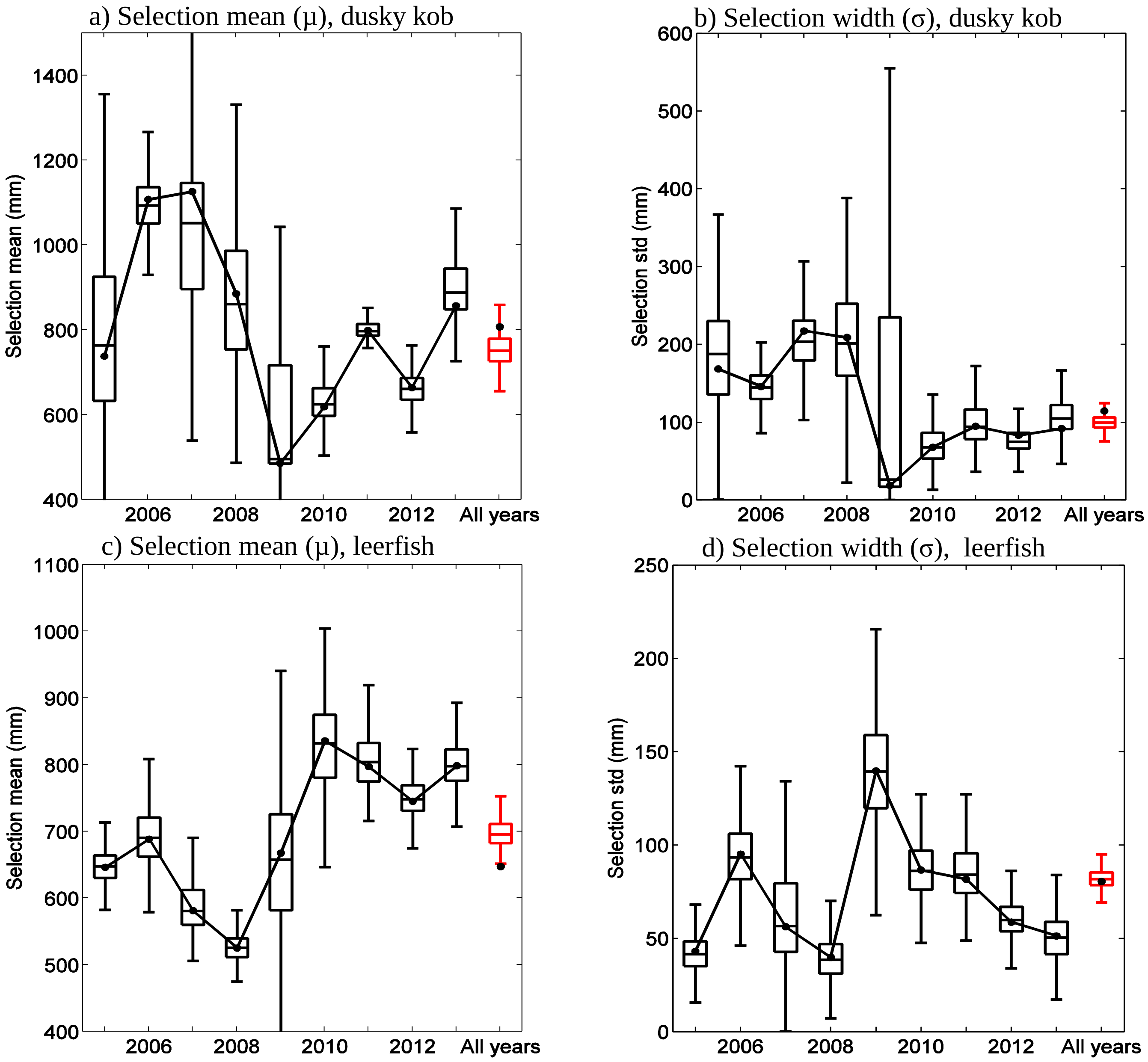

Figure E. Estimation of selectivity parameters based on equilibrium parameter estimation approaches.

Estimates of the inflection point and width of the selectivity ogive, μ_S and σ_S respectively, over 9 years for dusky kob (*A. coronus*) (a,b) and leerfish (*L. amia*) (c,d). Lines show model predictions using the original dataset, whereas box plots display distributions computed from 1000 random resamplings with replacements of individual fish lengths. Box plots show the median (center line), interquartile range (boxes), and limits beyond which data is considered to be an outlier (i.e., ~3 standard deviations for normally distributed data; whiskers). Results for two different parameter estimation approaches are presented in each panel: for approach i, nine yearly boxplots are given, starting with 2005 to the left of each panel; and for approach ii, a single boxplot is given to the right of each panel.

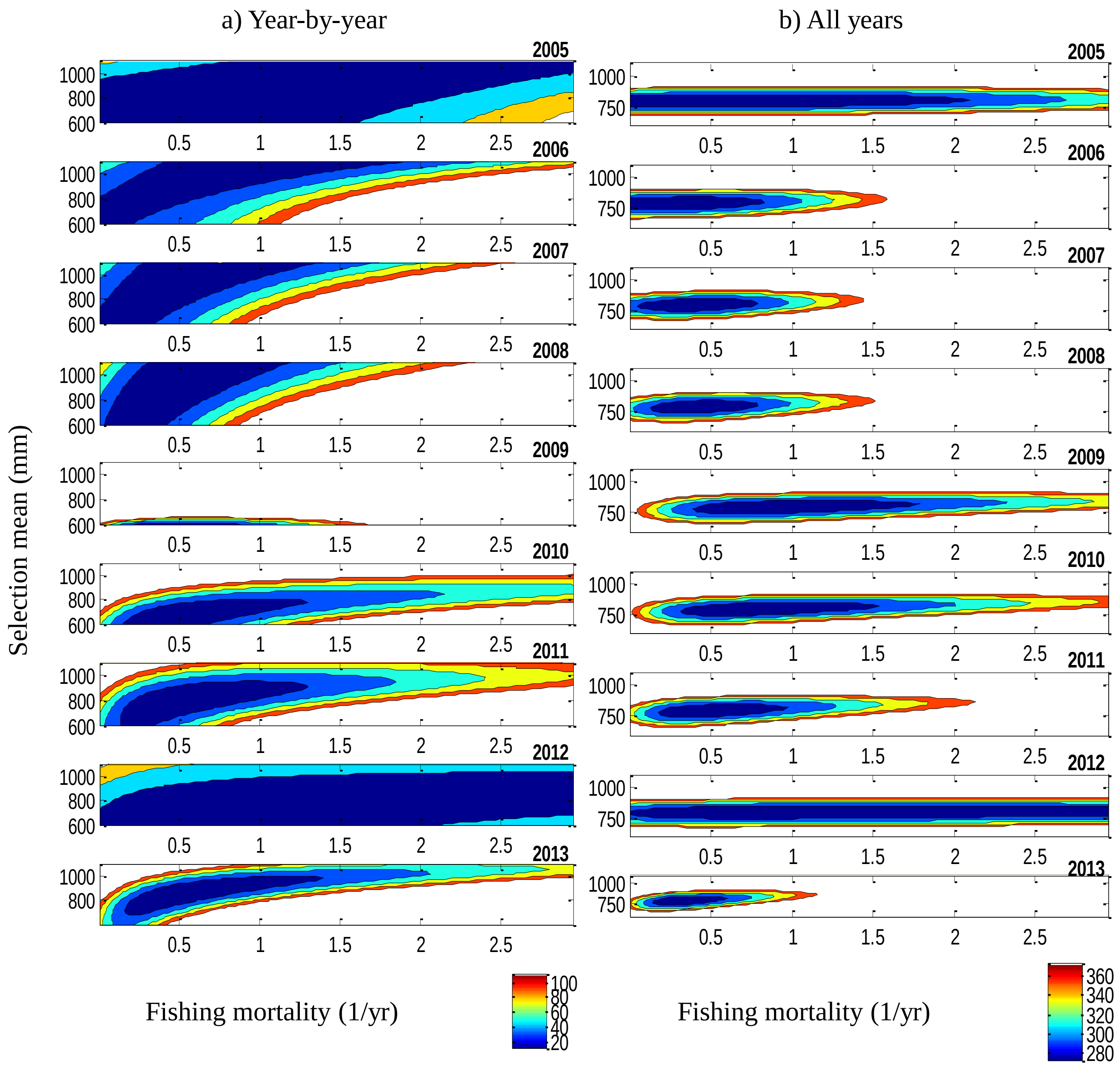

Figure F. Likelihood surface profiles for dusky kob over 9 years.

Exploration of KL divergence values as a function of the value of the inflection point of the selectivity ogive μ_S and the fishing mortality F calculated for each year for dusky kob (*A.coronus*). In all panels, fishing activity is assumed to start in 2005 (i.e., the "n-fishing-years" hypothesis is used). Results for parameter estimation approaches iii and iv are shown: year-by-year selectivity (a) and constant selectivity (i.e., "all-years"; b). For all years, 100 fishes were caught except for 2012 (i.e. 15 fishes) and for 2013 (i.e. 180).

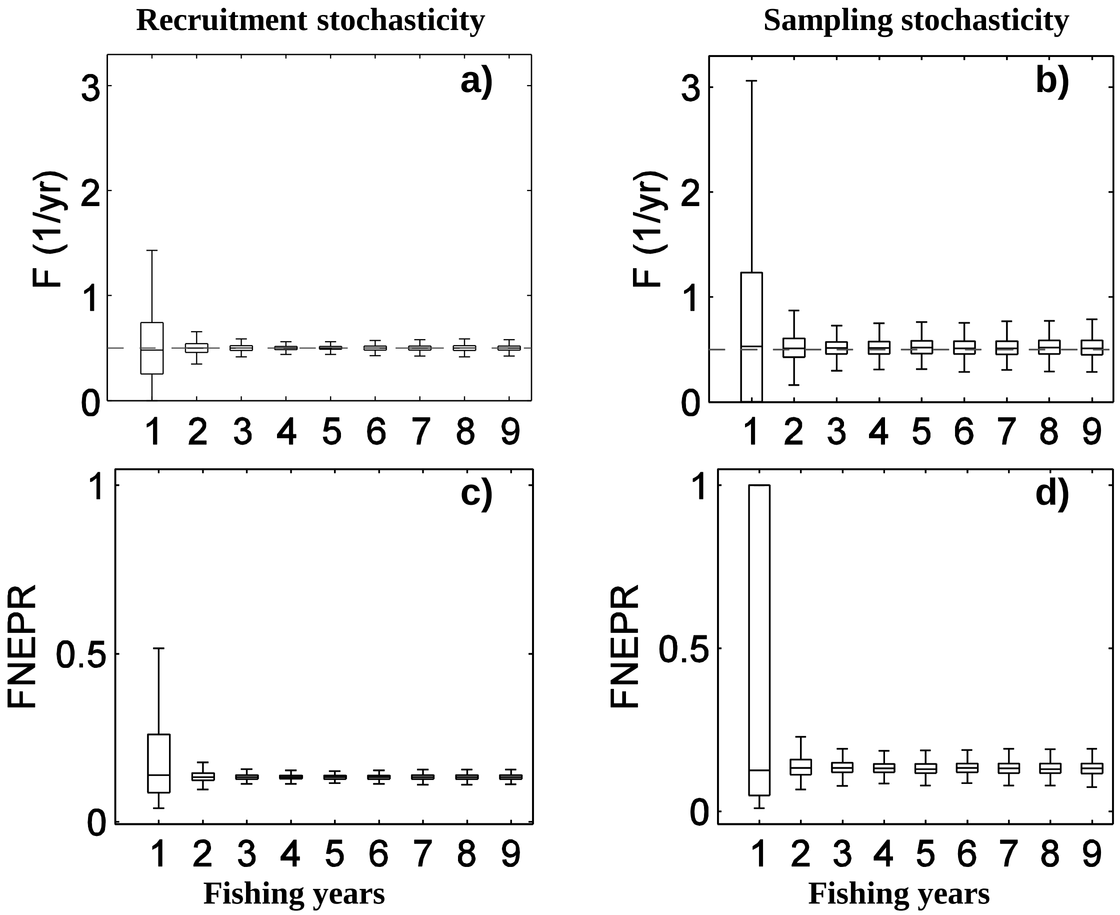

Figure G. F and FNEPR uncertainty estimates from simulations 3 and 4 for leerfish.

Uncertainty in F and FNEPR for leerfish (*L. amia*) from simulated size distributions from simulations with recruitment stochasticity (a, c; simulation 3) and sampling stochasticity (100 fishes) (b, d; simulation 4). Results for parameter estimation approache iv are shown. Box plots show the median (center line), interquartile range (boxes), and limits beyond which data is considered to be an outlier (i.e., ~3 standard deviations for normally distributed data; whiskers).

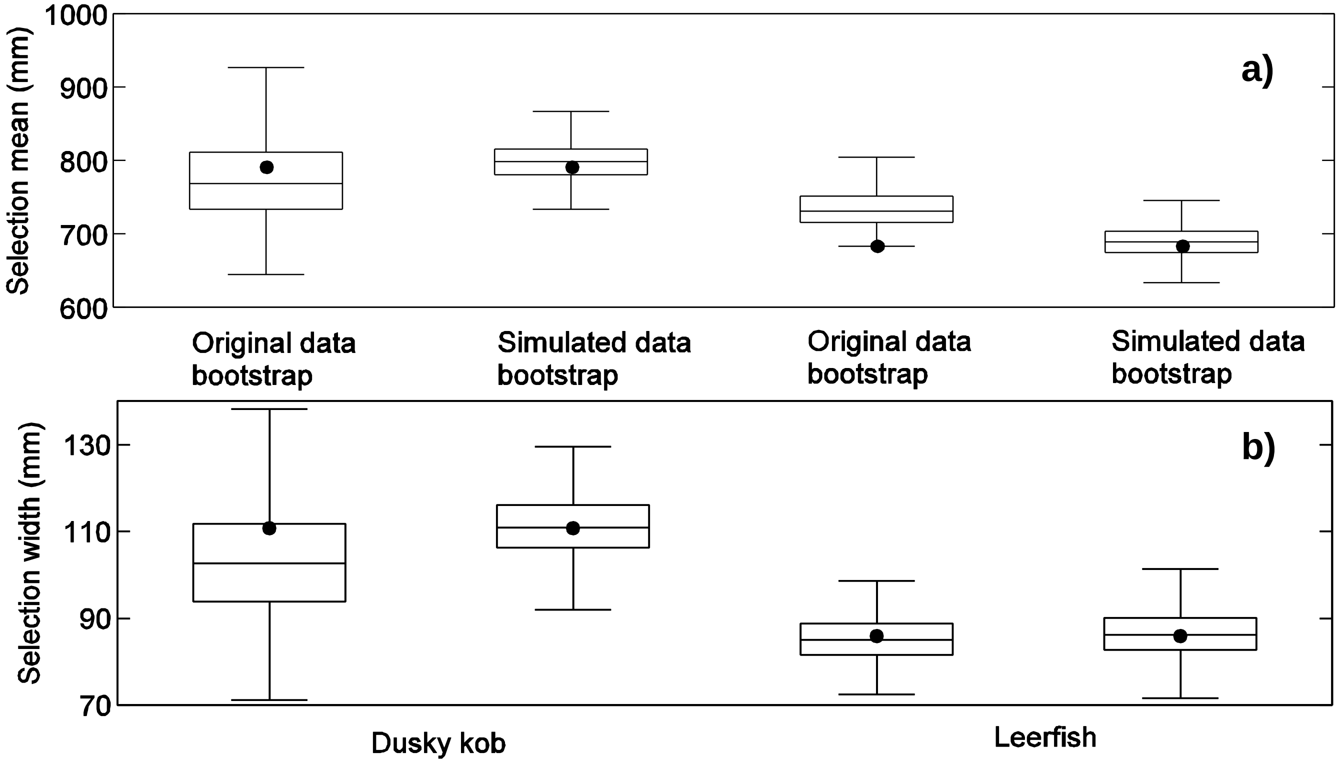

Figure H. Estimation of uncertainty of selectivity parameters.

Uncertainty of selectivity parameter estimates, i.e. of the inflection point μ_S (a) and the width of the selectivity ogive σ_S, for dusky kob (*A. coronus*) and leerfish (*L.amia*) for parameter estimation approach iv, i.e. constant selectivity assumption. Results are computed from 1000 bootstraps on Angolan size distributions, and from 1000 bootstraps on simulated size distributions. The true estimated value for dusky kob are μ_S = 791.93 and σ_S = 110.71 mm TL and for leerfish μ_S = 684.25 and σ_S= 85.88 mm FL.
