# Supplementary material for: Data-Limited Population-Status Evaluation of Two Coastal Fishes in Southern Angola Using Recreational Catch Length-Frequency Data
Source: PLoS One. 2016 Feb 1;11(2):e0147834. doi: 10.1371/journal.pone.0147834 (PMC4734550; doi:10.1371/journal.pone.0147834)
Supplement: S3 File — (PDF) [file pone.0147834.s003.pdf]

Data-limited population-status evaluation of two coastal fishes in southern Angola using recreational catch length-frequency data. Beckensteiner, J., Kaplan, D.M., Potts, W. M., Santos, C.V., O'Farrell, M.R.

S3 File

Table A. Data availability by catch size classes per year and per species.

Argyrosomus conous

Catch size bins	2005	2005	2005	2005	2005	2005	2005	2005	2005
0	0	0	0	0	0	0	0	0	0
50	0	0.00	0.00	0.00	0.00	0.00	0	0	0
100	0	0.00	0.00	0.00	0.00	0.00	0	0	0
150	0	0.00	0.00	0.00	0.00	0.00	0	0	0
200	0	0.00	0.00	0.00	0.00	0.00	0	0	0
250	0	0.00	1.35	0.00	0.00	0.00	0	0	0
300	0	0.00	0.00	0.00	0.00	0.00	0	0	0
350	0	0.00	0.00	0.00	0.00	0.00	0	0	0
400	0	0.00	1.35	1.23	1.85	0.00	0	0	0
450	1	0.00	2.70	3.09	3.70	2.94	0	0	1
500	3	1.34	4.05	4.32	22.22	5.88	12.5	0	0
550	2	1.34	2.70	8.64	16.67	11.76	0	2	7
600	3	2.14	5.41	4.94	1.85	11.76	0	3	7
650	5	4.81	10.81	7.41	9.26	8.82	0	1	9
700	4	5.35	2.70	8.02	9.26	23.53	0	3	15
750	8	4.28	4.05	11.73	7.41	5.88	37.5	3	17
800	3	4.55	5.41	3.70	3.70	5.88	12.5	2	26
850	4	4.55	9.46	7.41	11.11	8.82	0	0	21
900	5	9.36	6.76	5.56	3.70	5.88	12.5	0	24
950	8	12.83	12.16	7.41	1.85	2.94	25	0	22
1000	4	11.76	1.35	7.41	5.56	2.94	0	1	7
1050	8	12.30	8.11	6.79	0.00	0.00	0	0	15
1100	11	9.36	10.81	3.70	0.00	0.00	0	0	4
1150	7	5.88	5.41	5.56	1.85	2.94	0	0	4
1200	6	5.61	0.00	1.23	0.00	0.00	0	0	1
1250	4	2.41	4.05	1.85	0.00	0.00	0	0	0
1300	3	1.34	1.35	0.00	0.00	0.00	0	0	0
1350	3	0.53	0.00	0.00	0.00	0.00	0	0	0
1400	3	0.27	0.00	0.00	0.00	0.00	0	0	0
1450	3	0.00	0.00	0.00	0.00	0.00	0	0	0
1500	2	0.00	0.00	0.00	0.00	0.00	0	0	0
1550	0	0	0	0	0	0	0	0	0
Total	100	100	100	100	100	100	100	15	180

Lichia Amia

Catch size bins	2005	2006	2007	2008	2009	2010	2011	2012	2013
200	0	0	0	0	1	0	0	0	0
300	0	10	1	8	12	0	1	0	0
400	0	19	1	24	17	1	4	1	0
500	10	16	7	147	30	5	1	5	0
600	32	63	20	135	21	15	11	7	5
700	59	115	17	71	39	28	24	34	20
800	21	75	20	50	16	35	9	29	31
900	10	30	16	13	4	19	1	4	12
1000	7	19	18	3	0	3	0	3	3
1100	1	5	2	1	1	0	0	1	0
1200	0	0	0	0	0	0	0	0	0
Total	140	352	102	452	141	106	51	84	71

Table B. Sensitivity analysis of biological input parameters on F estimates for dusky kob (*A. coronus*).

Years	2005	2006	2007	2008	2009	2010	2011	2012	2013
L_∞	2.46	2.70	3.40	4.50	12.57	10.92	7.71	14.27	5.18
k	2.14	1.91	2.40	3.18	9.25	7.98	5.63	10.12	3.71
t_0	1.26	1.07	1.37	1.86	5.54	4.75	3.39	5.91	2.19
CV L_∞	1.14	0.87	1.15	1.56	4.76	4.05	2.84	5.08	1.83
M	-1.10	-0.77	-0.98	-1.31	-3.77	-3.20	-2.29	-3.93	-1.53
b	0.00	0.00	0.00	0.00	0.00	0.00	0.00	0.00	0.00
μ_{mat}	-10.00	-1.79	0.63	2.24	8.68	7.62	4.36	10.47	2.48
σ_{mat}	0.00	0.00	0.00	0.00	0.00	0.00	0.00	0.00	0.00
